# Effectiveness of Communication Competence in AI Conversational Agents for Health: Systematic Review and Meta-Analysis

**DOI:** 10.2196/76296

**Published:** 2025-11-03

**Authors:** Jiaqi Qin, Yuanfeixue Nan, Zichao Li, Jingbo Meng

**Affiliations:** 1 School of Communication, The Ohio State University, 154 N Oval Mall, Columbus, OH, 43210, United States, 1 5173034870; 2 Division of Infectious Diseases, Department of Medicine, Heersink School of Medicine, The University of Alabama at Birmingham, Birmingham, AL, United States; 3 Center for AIDS Research, The University of Alabama at Birmingham, Birmingham, AL, United States; 4 TH Chan School of Public Health, Harvard University, Boston, MA, United States

**Keywords:** conversational agent, AI, communication competence, health, systematic review, meta-analysis

## Abstract

**Background:**

With advancements in artificial intelligence and large language models, researchers and designers have increasingly focused on enhancing the conversational capacity of health-related conversational agents (CAs). Communication competence, a key concept in interpersonal communication influencing relational and health outcomes, has been extended to human-machine communication to emphasize the CAs’ ability to demonstrate appropriate communicative behaviors in managing relationships with humans.

**Objective:**

This review aims to summarize the operationalization of communication competence in health CAs and assess its impact on 4 primary outcomes: users’ evaluations of CA, use of CA, psychological outcomes, and health outcomes.

**Methods:**

A systematic literature search was conducted in 7 databases (ACM Digital Library, APA PsycInfo, Communication and Mass Media Complete, ProQuest Dissertations & Theses, Scopus, Web of Science Core Collection, and PubMed). Studies were included if they adopted experimental designs to manipulate CAs’ communication competence in health-related conversations, recruited human participants, and reported at least 1 relevant outcome. Risk of bias was assessed using the revised Cochrane risk-of-bias tool. The systematic review summarized commonly used communication competence strategies. Three-level random-effects meta-analytic models were used to estimate pooled effect sizes for 4 primary outcomes. Moderator analyses were conducted to assess whether effect sizes varied across publication year, participants’ average age, type of interaction with CAs, health topics, and publication outlet.

**Results:**

Of the 8309 identified papers, 31 independent experimental studies were included in the systematic review. Eleven strategies were identified to enhance CAs’ communication competence: empathetic response, contingency, humor, small talk, emotional expressiveness, self-disclosure, personalization, social etiquette, explanation, open-ended questions, and partnership. Of the 31 studies, 25 met the criteria for meta-analysis, which involved 4525 participants with a mean age of 29.7 (SD 9.2) years. The meta-analytic findings showed that communication competence has a significant small-to-medium effect on users’ evaluations of CAs (Hedges *g*=0.45, 95% CI 0.24‐0.66) and psychological outcomes (Hedges *g*=0.49, 95% CI 0.19‐0.78). The effect sizes on the use of CA (Hedges *g*=0.11, 95% CI −0.05 to 0.26) and health outcomes (Hedges *g*=0.18, 95% CI −0.13 to 0.50) are not significant. Moderator analyses showed that the effects remain stable across participants’ age, type of interaction, and health topics.

**Conclusions:**

This review highlights communication competence as a critical component in the design of health care CAs, particularly in improving users’ evaluations and psychological outcomes. However, the limited number of studies examining health outcomes restricts the robustness of its effectiveness on this outcome. Future research is encouraged to directly evaluate the effects on tangible health outcomes.

## Introduction

### Background

Recent advances in large language models (LLMs) have attracted great attention in health care for their capability to understand natural language and generate high-quality content. Increasingly, LLMs are integrated into conversational agents (CAs) to improve medical consultations and health interventions [1-3]. CAs, which are software systems designed to interact with humans through natural language [4], are playing an increasingly important role in health applications, such as delivering psychotherapy and remote health care services, providing health education information, conducting clinical interviews, assisting with symptom diagnosis, and promoting health behaviors [5,6]. As these advanced technologies become more prevalent in health care and increasingly involve the use of personal information, researchers have dedicated greater attention to evaluating their potential benefits [7] as well as addressing emerging challenges such as privacy and data security [8].

Recent meta-analytic reviews confirm the effectiveness of CA-based interventions in reducing depression and anxiety and promoting healthy behaviors [9-11]. However, these studies primarily compare interventions with and without CAs, without isolating the impact of specific CA features. While some reviews have cataloged a range of chatbot features, from platform and modality to anthropomorphism [5,6,12], few offer an in-depth analysis of communication-specific features, despite their centrality in CA interactions and recent advancements driven by LLMs.

To address this gap, our review focuses on the relational communication capacity of health care CAs through the lens of communication competence, a foundational concept in interpersonal communication linked to emotional intelligence and relationship building [13]. A recent review [14] has attempted to examine the effectiveness of communication features of health care CAs by including CAs’ persuasive (eg, use of persuasive tactics) and relational (eg, showing empathy) communication capacity. To extend the prior work, our review will fully unpack the communication features that enhance CAs’ communication competence and examine their effectiveness across various outcomes of health care CAs.

### Communication Competence

Communication competence, which originally came from interpersonal communication, refers to a person’s ability to engage in appropriate communicative behaviors in order to manage interpersonal relationships [13]. Communication competence involves a set of communicative skills, such as engaging in self-disclosure, expressing empathy, using interaction management skills, conveying appropriate emotions, and exhibiting social relaxation [13,15]. In interpersonal communication, communication competence is closely related to various relational outcomes such as relational closeness [16] and relationship satisfaction and commitment [17,18]. The concept of communication competence has been extended to the context of human-machine communication to emphasize CAs’ communicative skills of facilitating appropriate communication and managing their relationship with human users [19]. Recently, various communication competencies have been incorporated into CAs, such as providing empathetic responses, demonstrating attentiveness, exhibiting politeness, using humor and social praise, and engaging in socioemotional dialogue and small talk [20-22].

Communication competence has long been recognized as essential in face-to-face health care contexts. Medical education and provider training emphasize skills such as active listening, demonstrating empathy, adapting to patients’ communication preferences, using plain language, and offering emotional support [23-25]. In patient-provider communication, both patients and providers highlight 2 primary aspects of the provider’s communication competence: effective information exchange (eg, explaining diagnosis and treatment, ensuring patient understanding, and using plain language to explain technical information) and relational development (eg, creating a warm and friendly environment, conveying care and concern, and providing emotional support) [26]. These competencies are linked to stronger patient-provider relationships and improved health outcomes, particularly in chronic disease management [27-30].

Given its importance, communication competence is increasingly embedded in health-related CAs, with features such as empathetic language, personalized responses, small talk, and adaptation to users’ communication styles [9,10,14]. However, there is limited systematic understanding of how communication competence has been conceptualized and operationalized in CA research. Filling this gap will contribute to the literature by identifying aspects of communication competence that have or have not been examined, which will improve our understanding of the effectiveness of communication competence. Therefore, the following research question (RQ) is proposed:

RQ1: How is communication competence operationalized in artificial intelligence (AI) CAs for health?

### Outcomes of CA Communication Competence

When examining the impacts of CAs within the health care context, user experience, psychological outcomes, and health outcomes are primary evaluation dimensions [5,6,10]. User experience, which emphasizes users’ perceptions and responses resulting from their interactions with CAs [31], usually includes user satisfaction, usability, perceived ease of use, perceived enjoyment, perceived helpfulness, working alliance, likeability, intention to use, and user engagement [5,10]. User experience is critical for the acceptance of new technologies in health care and contributes to the improvement in health outcomes [32]. In addition, communication-related factors, such as empathic communication, nonjudgmental interactions, and tailored feedback, have been identified as primary factors in fostering a positive user experience [10]. Therefore, we consider user experience as a key outcome category for communication competence in this review and further categorize it into 2 aspects: the evaluation of CA, which involves all aspects of users’ perceptions regarding CAs, and the use of CA, which emphasizes behavioral aspects such as actual engagement and intention to use CAs.

In addition to user experience, psychological outcomes are also influenced by communication competence. For example, one study found that patients of physicians who demonstrated more sensitivity, understanding, empathy, and positivity during the medical visit experienced greater reduction in emotional distress [33]. A recent meta-analysis has also found that physicians’ communication competence is positively associated with patients’ perceived psychosocial adjustment [34]. Given the importance of communication competence in health and interpersonal communication, we consider psychological outcomes to be an important outcome category influenced by CA communication competence.

Health outcomes are another critical consideration. A meta-analysis found a significant positive relationship between physician’s communication competence and patient adherence to treatment [35]. Another study found that patients who rated their physicians as conveying more positive regard, openness, and empathy during medical encounters experienced greater improvement in pain impairment, intensity, and frequency [36]. The specific outcomes examined depend on the goals of the CA application, such as treatment adherence, pain-related impairment, and improvement in healthy eating and physical activity [5,6,14,37]. Given their relevance, health outcomes form the last outcome category in our review.

Although research on the communication competence of CAs has suggested that it influences user experience, psychological outcomes, and health outcomes, empirical findings on its effects remain mixed. Regarding users’ evaluations of CAs, one study found that chatbots conveying empathy were perceived as more supportive than those providing advice only [38]. However, another study found no significant differences in therapeutic alliance or perceived empathy between chatbots with multiple communication skills and those without [39]. Findings on psychological outcomes and health outcomes are also inconsistent. Some studies reported that chatbots providing more supportive feedback enhance users’ perceived emotional validation, fostering a greater sense of understanding and acknowledgment of their emotions [40]. However, other studies found no significant differences in emotional validation between users interacting with high- and low-contingent chatbots [41]. For health outcomes, one study found that participants who interacted with CAs providing empathetic responses reported lower chronic pain intensity after the intervention [37], while other research showed no effects of CA communication competence on the user’s motivation to quit smoking [39] or intention to cook recommended healthy recipes [42]. To assess the overall effectiveness of communication competence of CAs in health care, the second goal of this review is to synthesize these mixed findings on user experience and psychological and health outcomes through a meta-analysis.

RQ2: What is the overall effect of enhancing CAs’ communication competence on users’ (1) evaluation of CAs, (2) use of CAs, (3) psychological outcomes, and (4) health outcomes?

### Potential Moderators of the Effectiveness

Considering the mixed findings in previous empirical studies, it is important to identify the moderators to provide a nuanced understanding of the effectiveness of communication competence in CAs for health. Publication year may serve as an important moderator because there might be a natural user maturation with new technologies over time. In the early years, machines were generally perceived as lacking the capability to feel and sense [43,44]. When machines exhibited feelings, they would induce feelings of uncanniness [45]. However, as emotionally expressive CAs have become more common, users may have developed greater acceptance, reducing feelings of unease and increasing receptivity to communicative features.

Age is another potential moderator, as attitudes and experiences with AI vary across age groups. Previous studies have indicated that compared with young people, older adults tend to hold more negative attitudes toward AI technology [46,47]. Older people have also been reported to have less knowledge and experience about AI [46,48]. Although communication competence may enhance CAs’ anthropomorphism [49] and thus should elicit positive effects on relevant outcomes, it is also possible that older adults’ general fear and anxiety about the AI technology will wash away the effect of communication competence.

In addition, the type of interaction with CAs may moderate the effectiveness of communication competence. In human-machine communication research, experimental studies generally use three methods to create interactions with agents: (1) prerecorded materials, which display the screenshots or video recordings of the conversation between human users and CAs; (2) the Wizard of OZ technique, where participants believe that they are interacting with a CA that is actually controlled by human experimenters remotely; and (3) real CAs, either created by researchers or commercially available [50]. While prerecorded materials allow researchers to easily manipulate desired CA characteristics in a large sample, they lack authentic interaction between participants and CAs [50], which can result in less intense user experiences [38]. Although the Wizard of Oz method involves user interaction with CAs, the smoothness of the conversation may lead participants to suspect the agency of the conversational partner. It remains unclear whether the effect sizes examined with this approach are similar to those using real CAs.

We also consider health topics as a potential moderator, given the variation in effect sizes across different medical conditions in previous reviews on health care professionals’ communication competence [35]. Health care CAs have been applied in various health contexts, such as providing mental health services, conducting disease diagnosis, offering disease treatment, and promoting healthy behavior change [6]. In health contexts such as mental health services, where users desire more emotional support and long-term companionship, the communication competence of CAs may have a stronger influence. In contrast, in health contexts where the provision of accurate health information and practical health advice is prioritized, the communication competence of CAs may be less effective. Based on the discussion about moderators, our third RQ intends to explore the role of 4 potential moderators on the effectiveness of CAs’ communication competence:

RQ3: Is the effectiveness of CAs’ communication competence moderated by (1), publication year, (2) participants’ average age, (3) type of interaction with CAs, and (4) health topics?

## Methods

### Literature Search

The literature search was conducted in 7 databases: ACM Digital Library, APA PsycInfo, Communication and Mass Media Complete, ProQuest Dissertations & Theses, Scopus, Web of Science Core Collection, and PubMed. Given that CAs are an emerging technology, we adopted a keyword-based search strategy based on the approach used in previous reviews on the effectiveness of CAs [9,10,14]. To ensure broad coverage of health-related topics and outcomes, no keywords or MeSH terms on specific health conditions or diseases were included in the search terms at the literature search stage. Instead, health relevance was ensured during the screening stage by applying an inclusion criterion focusing on health-related conversations involving CAs. Specifically, we combined 3 sets of search terms to search the title and abstract of the literature: variants and synonyms of CAs (eg, conversational agent*, chatbot*, conversational system*, conversational AI, and dialog* system*), communication competences (eg, communication skill*, conversational capability*, social skill*, and conversational style*), and experimental designs (eg, experiment*, randomized controlled trial, and random* assign*). Literature published in English between January 1, 2003, and December 31, 2023, was included in the search. The complete list of search queries can be found in Multimedia Appendix 1. The reference lists of the included papers were also searched to identify any additional literature.

### Inclusion and Exclusion Criteria

Table 1 summarizes the inclusion and exclusion criteria following the PICOS (Population, Intervention, Comparison, Outcome, Study design) framework.

**Table 1.** Summary of inclusion and exclusion criteria^a^.

Variable	Inclusion criteria	Exclusion criteria
Population	Human participants of any demographics and health conditions.	Studies without human participants (eg, simulations).
Intervention	Studies that manipulated the communication competence of a nonembodied and unconstrained CA^b^ in conversations about health-related topics.	CAs with physical or virtual embodiments (eg, embodied CAs, robots, or virtual avatars). Constrained CAs that restrict user interaction to predefined options. Studies applied CAs in nonhealth topics (eg, marketing and education). Studies manipulated CAs’ features unrelated to communication competence, such as appearance, identity, and gender.
Comparison	Studies with a control group using CAs with no or low levels of communication competence, or without a comparison (eg, pre-post design).	Studies that use standard care or other technologies that did not involve CAs.
Outcome	Measure at least 1 outcome related to: Evaluations of CA Use of CA Psychological outcomes Health outcomes	Studies that report only technical performances of developed methods or algorithms.
Study design	Experimental studies.	Reviews, qualitative studies, survey studies, or other nonempirical studies.

a In addition to the PICOS criteria listed in the table, we had an additional eligibility criterion that studies needed to be complete research papers written in English and published in peer-reviewed journals, conference proceedings, dissertations, or theses. For meta-analysis, included studies needed to have a control group using the corresponding conversational agents with no or low levels of communication competence and reported sufficient information to calculate effect sizes.

b CA: conversational agent.

#### Population

Studies involving human participants of any demographics or health condition were eligible for the review. This broad scope is to include studies targeting a diverse range of populations to better understand the effectiveness of the communication competence of CAs. Studies that did not recruit human participants were excluded.

#### Intervention

Eligible studies needed to examine nonembodied CAs that accept unconstrained natural language input. We excluded studies on CAs with physical or virtual embodiments, such as embodied CAs, robots, or virtual avatars, which involve multimodal interaction with human users and use various nonverbal cues (eg, facial expressions, body movements, and eye contact) in the conversation. This exclusion was made to ensure a focus on the conversational capability of CAs and avoid distractions of visual embodiment and nonverbal cues in the evaluation of communication competence. Another type of CA we excluded is CAs that restrict user interaction to predefined options through clicking and pointing, without allowing natural language input from users. Considering the rapid development in LLM and the increasing conversational capability of CAs, we want to focus on CAs that accept unconstrained human language [5].

In addition, eligible studies needed to apply CAs in health-related topics (eg, medical consultation, therapy delivery, health behavior coaching, or discussion of health issues) and manipulate the communication competence of CAs within the conversation.

#### Comparison

Eligible studies needed to either include a control group using the corresponding CAs with no or low levels of communication competence or adopt a design without a comparison group (eg, pre-post designs). Studies that adopted a comparison group using standard care or other technology-assisted interventions that did not use CAs were excluded.

#### Outcome

Eligible studies needed to measure at least 1 outcome variable regarding the evaluation of CA, use of CA, psychological outcomes, or health outcomes. Studies were excluded if they reported only the technical performance metrics of algorithms or developed methods. Research proposals that did not report any outcomes were also excluded.

#### Study Design

Eligible studies needed to adopt an experimental study design to evaluate the effectiveness of CAs’ communication competence. Observational and qualitative studies were excluded. Nonempirical papers, such as reviews and theoretical articles, were also excluded.

#### Additional Criteria

Eligible studies were required to be complete research papers written in English and published in journals, conference proceedings, or as a dissertation and thesis. To be included in the meta-analysis, the papers must meet 2 additional criteria beyond those listed in the PICOS framework. First, the experimental manipulation of communication competence needs to include at least 1 control group (ie, a condition that used the same CA but either lacked or exhibited a lower level of certain communication competence). Second, the papers need to report effect sizes or provide sufficient information to calculate effect sizes for relevant outcomes.

### Study Selection

All records identified through the literature search were first imported into Covidence (Veritas Health Innovation Ltd), a software for managing systematic reviews, to remove duplicates. Title and abstract screening was conducted by 3 reviewers (JQ, YN, and ZL). Prior to screening, they met to review the criteria and procedure for screening and independently screened a subset of 30 records. The average agreement for this subset was 0.98, and disagreements were resolved through discussion. The remaining records were then screened independently by the 3 reviewers. For full-paper screening, the same 3 reviewers read the full text of each paper to assess their eligibility. After 2 rounds of training and discussion, the average agreement reached 100% on a subset of full-text records.

### Risk of Bias Assessment

Risk of bias was assessed using the revised Cochrane tool [51], which evaluates 5 domains: the randomization process, deviations from intended interventions, missing outcome data, measurement of the outcome, and selection of the reported result. The overall risk of bias for each study was determined based on the judgments for all 5 domains. Studies were evaluated independently by 2 reviewers (JQ, ZL). Disagreement between the 2 reviewers was resolved through discussion.

### Data Extraction

Eligible papers were read by 4 reviewers (JQ, YN, ZL, and ZY) to extract information about study design, sample characteristics, CA features, manipulated communication competence, and outcome variables. Information on study design included the publication year, publication outlet (ie, journal publication, conference proceeding, or thesis and dissertation), experiment setting (ie, laboratory, web-based, or field experiment), experiment design (eg, between-subject or within-subject design), and type of interaction (ie, designed CAs, prerecorded materials, or Wizard of Oz method). Sample characteristics were sample size, female percentage, average age, race percentage, education, use of student-only sample, and country. CA features included conversation modality and health topics. For health topics, 2 researchers (JQ and JM) reviewed eligible papers to inductively identify the following categories: teletriage, medical information and advice provision, health behavior promotion, and mental health.

Regarding communication competence, we extracted the manipulated communication competence variables and their operationalization. The extracted communication competence was then categorized using a mixed approach. We adopted certain categories from the framework proposed by Rubin and Martin [13] and modified some categories to better fit the extracted competence. In addition, new categories were identified inductively for the communication competence not covered in the existing framework. For the outcome variables, we extracted all outcomes examined in the study because we are interested in the effectiveness of communication competence on different types of outcomes. The extracted outcome variables were then categorized into 4 types: evaluations of CA, use of CA, psychological outcomes, and health outcomes. Manipulation check variables were excluded from the outcome variables.

Hedges *g* was used to synthesize effect sizes in this meta-analysis because the sample size of some included papers was small. As Cohen *d* may overestimate the effect size when calculated from small samples, Hedges *g* is recommended if the sample size of the synthesized study is smaller than 20 [52]. The mean, standard deviation or standard error, and sample size of the outcome variables in the control and treatment groups were extracted to calculate the effect size. When this information was not available, we first converted other statistics (ie, *t* test statistics, *F* statistics, zero-order correlations, and odds ratios) reported in the paper to Cohen *d* and then to Hedges *g* using the corresponding formulas provided in Multimedia Appendix 2 [52-54]. If the authors did not provide the sample size for each experiment condition, an equal sample size was assumed. The direction of the effect size was determined based on the nature of the outcome variables. For example, if the outcome is undesired, such as privacy concerns, a higher value in the treatment group compared with the control group indicates a negative relationship.

Four reviewers were trained for data extraction. The agreement between reviewers was calculated using the intraclass correlation coefficient for continuous variables and the percentage of agreement for categorical variables. The average agreement ranged from 0.80 to 1 and all disagreements were resolved after discussion.

### Statistical Analysis

To assess the effectiveness of CAs’ communication competence on 4 outcomes, we conducted separate meta-analyses for users’ evaluations of CA, use of CA, psychological outcomes, and health outcomes. Because we extracted all relevant outcomes reported in each study, some studies contributed multiple effect sizes to 1 outcome category. To handle the dependence among multiple effect sizes from the same study, we adopted a multilevel approach, as recommended by Van den Noortage and colleagues [55], which has been applied in several recent meta-analyses to address this issue [56,57]. This approach builds multilevel models to account for variations of effect sizes from 3 levels: the sampling variance of each effect size (level 1), variances between effect sizes within the same study (level 2), and between-study variations (level 3) [55]. Specifically, the level 2 variance component enables researchers to model the dependence among multiple effect sizes from the same study [58]. We built four 3-level meta-analytic models using the *metafor* (version 4.6.0) package in R software (version 4.4.0; R Foundation for Statistical Computing), following the step-by-step guideline by Assink and Wibbelink [59]. The random-effects model was used for the analysis because it assumes that the effect sizes of the included studies were drawn from a distribution of true effect sizes of the population of studies [54]. *Q* statistics were calculated to assess the heterogeneity of effect sizes. For overall effect sizes with significant heterogeneity, moderators of interest were added to the multilevel model separately, and omnibus tests were conducted to assess the significance of the moderation effects [59].

## Results

### Study Selection

The initial literature search identified 8309 papers. After removing duplicates, 5441 papers remained for title and abstract screening. We applied the inclusion criteria for the systematic review for this round of screening. Of the 5441 papers, 4575 were excluded for the following reasons: (1) 1762 did not study CAs or studied CAs outside the scope of this review; (2) 1819 did not use an experimental design; (3) 859 did not manipulate communication competence in their experiments; (4) 29 did not measure relevant outcome variables; (5) 45 were duplicate papers identified by comparing titles, authors, and study details (eg, study design and sample characteristics); (6) 59 were nonresearch papers (eg, corrections and introductions to proceedings); and (7) 2 were not published in English. Following this screening stage, 866 papers were retained for full-text retrieval, with 849 retrieved successfully.

Of the 849 retrieved papers, 825 were excluded for the following reason: (1) 194 did not study CAs within the scope of this review, (2) 392 did not apply CAs in health-related domains, (3) 111 were not experimental studies, (4) 109 did not manipulate CAs’ communication competence, (5) 1 paper was not written in English, and (6) 18 were not complete research papers (eg, extended abstract and work in progress). This screening stage resulted in 24 papers included in the systematic review. In addition, 16 papers identified from the reference lists of the 24 included papers were retrieved for screening, and 7 of them met the inclusion criteria. In total, 31 papers were included in the systematic review.

**Figure 1.**
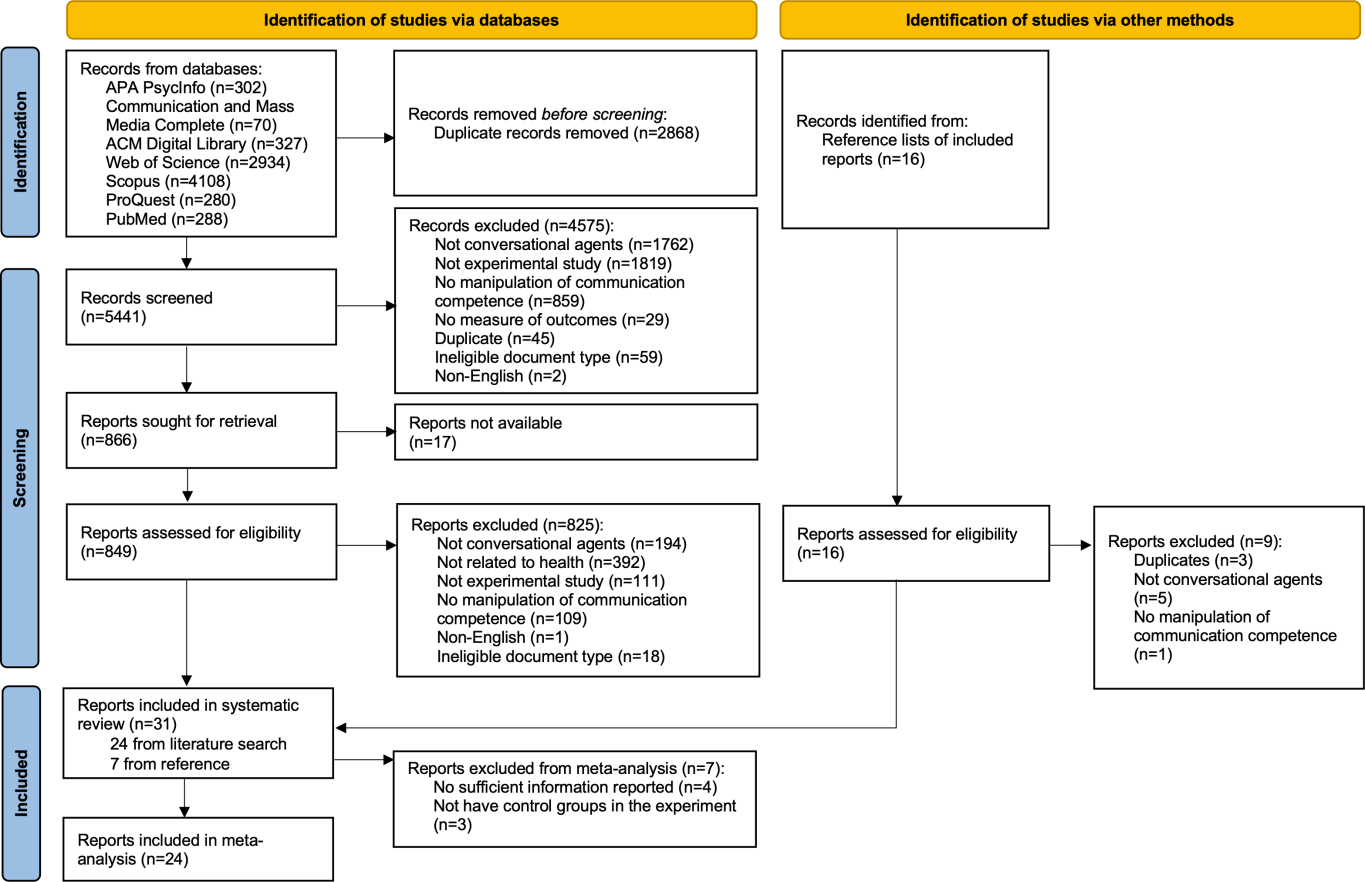
PRISMA (Preferred Reporting Items for Systematic Reviews and Meta-Analyses) flow diagram for systematic review and meta-analysis.

Two additional criteria were applied to these 31 papers to assess their eligibility for the meta-analysis. Among them, 4 papers were excluded due to insufficient information to calculate effect size despite attempts to contact the authors, and 3 were excluded for lacking control groups regarding the manipulation of communication competence. Consequently, a total of 24 papers were included in the meta-analysis. Figure 1 shows the complete process of literature identification and screening.

### Systematic Review

#### Description of Included Studies

A total of 31 research papers were included in the systematic review, resulting in 31 independent studies. Research papers that analyzed data from the same study [60-63] were treated as 1 single independent study. The papers were published between 2008 and 2023, with the majority published since 2020 (n=25). Of the 31 included studies, 21 used a between-subjects experimental design, 5 adopted a within-subject design, 3 used mixed design, and 2 were quasi-experiments with a single-group pretest-posttest design. In total, these studies involved 4850 participants from multiple countries, including the United States, China, Germany, Belgium, and the Netherlands. The average participant age across studies ranged from 14 to 51.9 years (mean 29.1, SD 9.1 years), and the percentage of female participants varied from 17.9% to 75.5% (mean 54.6%, SD 14.5%).

The key information extracted from the included studies is summarized in Multimedia Appendix 3 [38-42,49,60-84]. We extracted the characteristics of CAs adopted in the studies. Most studies presented a text-based CA (n=29), and only 2 studies [62-64] used a voice-based CA. Researchers presented interactions with CAs in different ways. Most studies designed a CA prototype and had participants interact with it (n=24). Three studies presented users with the prerecorded videos or scripts of the interaction between a user and a CA [38,49,65]. Four studies used the Wizard of Oz approach to have human experimenters control the CA to engage in the conversation with participants [41,66-68]. Of the 31 studies, CAs were applied across various health contexts, including teletriage services that collect patient information and refer patients to appropriate health care departments (n=1), medical information and advice provision (n=6), health behavior promotion (n=7), and mental health (n=17).

The studies evaluated a wide range of outcomes related to CAs and health. The most frequently assessed outcomes were variables related to the evaluation of CAs, which were reported in 26 studies, including overall evaluations (eg, trust, likeability, and satisfaction) and perceptions of specific CA characteristics (eg, perceived warmth, perceived competence, and perceived message supportiveness). Some studies also examined outcomes related to the use of CAs (n=13), such as the intention to use CAs [65,69] and the amount of information disclosure to CAs during the interaction [67,70]. Psychological outcomes were assessed in 15 studies, including positive and negative emotion [60,61,67], emotional validation [40,41], stress reduction [71,72], and the feeling of being supported [38]. Health outcomes were less commonly assessed, with only 5 studies addressing relevant variables. These studies primarily examined the intention to engage in recommended health behaviors (eg, intention to cook the recommended recipes and vaccination intention) and behaviors of opening for medical help (eg, disclosure to mental health professionals).

#### Operationalization of Communication Competence

Communication competence was operationalized in various ways. We identified 11 strategies used to enhance CAs’ communication competence: empathetic response, contingency, humor, small talk, emotional expressiveness, self-disclosure, personalization, social etiquette, explanation, open-ended questions, and partnership. Table 2 provides a summary of these 11 communication competence strategies and their operationalization across the studies.

**Table 2.** Operationalization of communication competence across studies.

Communication competence (number of studies), research papers	Operationalization
Empathetic response (n=18)	
Trzebiński et al. (2023) [73]	Express empathy and autonomy support by showing understanding of the users’ concerns, acknowledging their knowledge, supporting their autonomy regarding vaccination, showing interest in their situation, and offering comfort
He et al (2022) [39]	Incorporate reflective listening to acknowledge the user’s health concerns, affirm their points and efforts, validate their personal choice, and express compassion for their suffering
El Hefny et al (2021) [74]	Use empathetic expressions extracted from the “EmpatheticDialogues” dataset to approach the users in a friendly manner and create a bond
Gotthardt et al (2022) [64]	Use empathetic techniques such as mirroring, empathetic listening, cheering up, or calming in response to the user’s emotions and answers to the psychoeducational quiz
Ho (2018) [67]	Provide responses validating the conversational partner’s feelings
Meng and Dai (2021) [71]	Provide emotional support that communicates empathy, emotional validation, and encouragement to the conversational partner
Liu and Sundar (2018) (Study 1 and Study 2) [38]	Express sorry for the user in the beginning of each response (Sympathy) Recognize and acknowledge the user’s feelings and situations in the beginning of each response (Cognitive empathy). Express understanding of the user’s feelings in the response (Affective empathy)
Kraus et al (2021) [75]	Show empathetic reactions by expressing understanding of the user’s negative mood shared during the daily check-in
Rains et al (2020) [40], (2020) [76] Rains and High (2021) [72]	Provide responses that explicitly acknowledged and elaborated on the conversational partner’s feelings and offer suggestions on how to reframe negative affect
You et al (2023) [77]	Provide emotional support to the user by using caring language and encouragement as well as offering potential treatment suggestions
Beattie (2023) [66]	Provided highly person-centered responses that focus on the user’s emotional and stressors, including reflecting on, acknowledging, and confirming the challenges related to the topic, and emphasizing the normality of the stressful feeling
Pecune et al (2020) [42]	Use acknowledgments to show understanding of what users just said
Ghandeharioun et al (2019a) [60], (2019b) [61]	Acknowledging the user’s emotional state after receiving their mood report
Lin et al (2023) [78]	Show understanding of the users’ input and generate empathetic responses based on the user’s emotions
Contingency (n=5)	
Meng et al (2023) [41]	Repeat the conversational partner’s self-disclosure and refer to the conversational partner’s specific situations mentioned in the previous conversation
De Boni et al (2008) [79]	Preserve the records of previous conversations and refer back to the issues (eg, barriers and solutions) discussed in the previous conversations
He et al (2022) [39]	Summarize previous conversation
Liu et al (2022) [69]	Embed user’s personal information asked in the previous conversation (eg, gender, age, and eating and living habit) in the response when providing diagnostic suggestions
You et al (2023) [77]	Provide a personalized summary of the symptoms mentioned by the user in the previous conversation to explain the diagnosis
Humor (n=3)	
El Hefny et al (2021) [74]	Induce humor with GIFs to create a friendly and cheerful atmosphere
De Boni et al (2008) [79]	Use self-contained jokes at the end of each session and self-deprecation
Lopatovska et al (2022a) [62], (2022b) [63]	Tells jokes to the user
Small talk (n=5)	
Kraus et al (2021) [75]	Deal with casual topics such as music preferences, personality, daily feeling, daily plans, and weather
Kobori et al (2016) [80]	Generate small talk utterances by choosing an appropriate response from the database based on the preceding user response, such as utterances about food preference, taste, or fun facts about specific food
Pecune et al (2020) [42]	Engage in small talk in the introductory phase by asking the user’s name, whether they are doing good, typical food for dinner, and reasons behind their food choices
De Boni et al (2008) [79]	Incorporate small talk elements during the greeting, which became more personal over time
Lee et al (2020) [70]	Build a small-talk session to discuss topics such as favorite holidays and zoo experiences before moving on to sensitive questions
Emotional expressiveness (n=2)	
El Hefny et al (2021) [74]	Add positive emojis to the response to convey affection
Ghandeharioun et al (2019a) [60], (2019b) [61]	Use emotionally expressive texts and emojis to convey appropriate emotions in response to the user’s mood and during the delivery of interventions
Self-disclosure (n=6)	
Meng and Dai (2021) [71]	Respond with its past experiences, thoughts, and feelings related to stressful situations
Lee et al (2020) [70]	High self-disclosure: Reveal its deep feelings, thoughts, and experiences in the past in the small-talk session. Low self-disclosure: Revealed less frequent and less intense feelings, thoughts, and past experiences in the small-talk session
Pecune et al (2020) [42]	Disclose information about itself to the user (eg, their eating preference and habits) during the introductory and information-gathering phases
Mai et al (2021) [68], (2022a) [81], (2022b) [82]	Talk about its own experience and feelings in similar situations before asking about the user’s experiences
Personalization (n=1)	
Albers et al (2022) [83]	Provide persuasive messages considering the person’s current state (eg, barriers or resources), future states, and the effectiveness of persuasive strategies for other similar people. It also updated the persuasion algorithm based on the user’s involvement in the recommended activity
Social etiquette (n=4)	
Li et al (2023) [49]	Use expressions of self-introduction, greetings, farewells, thanks, and tips and advice
You et al (2023) [77]	Use friendly addresses and greetings in the beginning of the conversation
Pecune et al (2020) [42]	Use reciprocal appreciation to give feedback to the user’s response
Kraus et al (2021) [75]	Express appreciation to the user for sharing personal topics during the daily check-in
Explanation (n=4)	
Woodcock et al (2021) [65]	Provide an explanation for the disease diagnosis using input influence (mentioned 2 symptoms most likely to indicate the disease), social proof (stated that a large number of people with similar symptoms have the disease), or counterfactual explanation (provided the symptom most likely to change a clinician’s opinion)
Pecune et al (2020) [42]	Use personal opinions as explanations of recipe recommendation
You et al (2023) [77]	Explain the rationale of each probing question and potential diagnosis using verified medical information
Buzcu et al (2023) [84]	Generate health-related (ie, nutritional values, such as protein, calorie, vitamin, and cholesterol information) and preference-related (eg, user’s preferred cuisine and ingredient) explanations to users
Open-ended question (n=1)	
He et al (2022) [39]	Ask open questions to encourage people to reflect on the reasons for quitting smoking
Partnership (n=1)	
He et al (2022) [39]	Emphasize shared understanding between the user and the chatbot and ask for consent before moving on

Empathetic response, which emphasizes understanding others’ perspectives and responding from their positions [13], was the most commonly studied communication competence strategy (n=18). Studies primarily implemented this strategy in CAs by incorporating expressions of sympathy and compassion [38,39], acknowledging the user’s situation [66,76], understanding and validating their feelings [67,75], and offering comfort and encouragement [71,73].

Self-disclosure, which involves CAs revealing their personal information to users, is another frequently used communication competence strategy (n=6). It was mainly operationalized by having CAs disclosing different kinds of information to the user, such as their eating habits [42], recreational activities and interests [70], experiences and feelings related to exam anxiety [68,81,82], and stressful situations [71].

The third strategy is contingency (n=5), which builds CAs’ responses based on the messages sent by the user in the previous conversation [85]. By engaging in a back-and-forth conversation through contingent messages, CAs demonstrate their attentiveness and responsiveness to users’ input. Studies primarily implemented this strategy in 3 ways: offering a personalized summary of the prior conversation [39,77], embedding specific information mentioned by the user earlier in the conversation (eg, personal information [69] and stressful situation [41]), and preserving a record of conversation history to refer back to information discussed in previous conversations [79].

Other strategies to enhance CA communication competence include integrating a small-talk module before the main conversational session (n=5). This allows CAs to engage in casual conversations on various topics, such as daily feelings, favorite holidays, food preferences, and the weather. In addition, some studies incorporated social etiquette phrases to convey politeness and respect (n=4). For example, CAs were designed to express appreciation for users’ responses [42,75] and to use greetings, introductions, friendly terms of addresses, and farewells [49,77]. Offering explanations is another important strategy used to foster trust between the CA and users (n=4). CAs were equipped with different kinds of explanations to justify their recommendations and diagnosis decisions, such as user’s preference, social proof, and medical information.

The strategy of humor was used in 3 studies by adding jokes to the responses [62,63,79] and using humorous GIFs [74]. Emotional expressiveness demonstrated through emojis and texts to communicate feelings was used in 2 studies [60,61,74]. Other less frequently used strategies included providing personalized persuasive messages [83], enabling open-ended questions [39], and emphasizing a partnership between CA and the user [39].

### Meta-Analysis

The meta-analysis included 25 independent studies from 24 research papers published from 2008 to 2023. A total of 4525 participants were involved in these studies. Of the 25 independent studies, 15 were published in peer-reviewed journals, 8 were from conference proceedings, and 2 were dissertations. The average age of the participants ranged from 19.8 to 51.9 years (mean 29.7, SD 9.2 years).

The majority of the studies adopted a between-subjects experimental design (n=19), and 3 studies used a within-subjects design. Three studies with mixed designs all manipulated communication competence as a between-subjects factor. Regarding the experimental setting, most studies conducted web-based experiments (n=19). Three studies were field experiments, and 2 were conducted in laboratory settings. For the interaction type, most of the studies designed a CA to interact with participants (n=18), 4 studies used the Wizard of Oz method, and 3 studies presented participants with prerecorded materials.

#### Overall Effect Sizes of Communication Competence

Regarding evaluations and use of CAs, the results showed that increased communication competence of CA was significantly associated with more positive evaluations of CA, with the pooled effect size Hedges *g*=0.45 (*k*=105, *P*<.001, 95% CI 0.24-0.66). Based on Cohen guidelines [86], the magnitude of this effect size is small to medium. The average effect size of the communication competence of CAs on the use of CA was Hedges *g*=0.11. However, this effect size was not statistically significant (*k*=33, *P*=.17, 95% CI −0.05 to 0.26).

Regarding psychological outcomes, interacting with CAs that demonstrate enhanced communication competence was significantly associated with better psychological outcomes, with the effect size Hedges *g*=0.49 (*k*=26, *P*=.001, 95% CI 0.19-0.78). According to Cohen guidelines [86], the magnitude of this effect size is considered to be nearly medium. Regarding health outcomes, the overall effect size of CAs’ communication competence on health outcomes was Hedges *g*=0.18, which was not statistically significant (*k*=21, *P*=.26, 95% CI −0.13 to 0.50).

The *Q* statistics revealed significant heterogeneity in the variance of effect sizes for each of the 4 types of outcomes (evaluation of CA: *Q*_104_=632.52, *P*<.001; use of CA: *Q*_32_=83.43, *P*<.001; psychological outcomes: *Q*_25_=239.77, *P*<.001; and health outcomes: *Q*_20_=49.57, *P*<.001). The significant heterogeneity tests indicated that moderator analyses were needed to explain the variability for all 4 outcome variables.

#### Moderator Effects

To answer RQ3, a series of moderator analyses with each moderator included in the multilevel models separately were conducted for outcomes regarding evaluation of CA, use of CA, and psychological outcomes. We did not conduct moderator analyses for health outcomes because all 21 effect sizes were extracted from only 3 studies, which may lead to unreliable conclusions.

Coefficients of multilevel models with moderators for 3 outcomes are summarized in Tables 3-5, respectively. For evaluation of CA, no significant moderation effects were found for publication year (*b*=−0.02; *P*=.55), participants’ average age (*b*=−0.01; *P*=.41), type of interaction (*QM*_2_=0.28; *P*=.87), and health topic (*QM*_3_=3.08; *P*=.38).

Regarding the use of CA, publication year significantly moderated the effectiveness of communication competence (*b*=−0.04; *P*=.02). The results indicated that the overall effect size for the use of CA decreases by 0.04, with a 1-year increase in publication time. No significant moderation effects were found for other moderators (participants’ average age: *b*=0.00, *P*=.76; type of interaction: *QM*_1_=2.14, *P*=.14; and health topic: *QM*_2_=0.02, *P*=.99).

When it comes to psychological outcomes, publication year was the only significant moderator (*b*=−0.26; *P*=.004). The overall effect size for psychological outcomes decreased by 0.26, with a 1-year increase in publication year.

**Table 3.** Multilevel regression models with moderators for evaluation of conversational agents.

Models	B (SE)	95% CI	Omnibus test QM	*P* value
Model 1: Publication year			0.36	*.*55
Intercept	38.75 (63.72)	−86.15 to 163.64		
Publication year	−0.02 (0.03)	−0.08 to 0.04		
Model 2: Age			0.69	.41
Intercept	0.80 (0.43)	−0.06 to 1.65		
Participants’ average age	−0.01 (0.01)	−0.04 to 0.02		
Model 3: Type of interaction (reference: interacting with designed CAs^a^)			0.28	.87
Intercept	0.44 (0.14)	0.17 to 0.71		
Viewing prerecorded materials	−0.07 (0.30)	−0.67 to 0.52		
Wizard of OZ	0.13 (0.31)	−0.48 to 0.73		
Model 4: Health topic (reference: mental health)			3.08	.38
Intercept	0.59 (0.16)	0.28 to 0.90		
Teletriage	0.16 (0.48)	−0.77 to 1.10		
Promote health behavior	−0.46 (0.31)	−1.06 to 0.14		
Provide medical information and advice	−0.25 (0.25)	−0.73 to 0.23		
Model 5: Publication outlet (reference: journal publication)			0.19	.91
Intercept	0.43 (0.15)	0.14 to 0.72		
Conference proceeding	0.00 (0.25)	−0.49 to 0.49		
Thesis and dissertation	0.18 (0.41)	−0.63 to 0.98		

a CA: conversational agent.

**Table 4.** Multilevel regression models with moderators for use of conversational agent.

	B (SE)	95% CI	Omnibus test QM	*P* value
Model 1: Publication year			5.37	.02
Intercept	75.12 (32.38)	11.66 to 138.58		
Publication year	−0.04 (0.02)	−0.07 to −0.01		
Model 2: Age			0.09	.76
Intercept	0.16 (0.26)	−0.36 to 0.68		
Participants’ average age	0.00 (0.01)	−0.02 to 0.01		
Model 3: Type of interaction (reference: interacting with designed CAs^a^)			2.14	.14
Intercept	0.16 (0.09)	−0.01 to 0.33		
Wizard of OZ	−0.30 (0.20)	−0.70 to 0.10		
Model 4: Health topic (reference: mental health)			0.02	.99
Intercept	0.09 (0.11)	−0.12 to 0.31		
Promote health behavior	0.04 (0.24)	−0.43 to 0.50		
Provide medical information and advice	0.01 (0.31)	−0.60 to 0.62		
Model 5: Publication outlet (reference: journal publication)			1.95	.38
Intercept	0.08 (0.09)	−0.09 to 0.25		
Conference proceeding	0.22 (0.18)	−0.13 to 0.57		
Thesis and dissertation	−0.09 (0.21)	−0.50 to 0.32		

a CAs: conversational agents.

**Table 5.** Multilevel regression models with moderators for psychological outcomes*.*

	B (SE)	95% CI	Omnibus test QM	*P* value
Model 1: Publication year			8.20	.004
Intercept	533.22 (186.03)	168.62 to 897.83		
Publication year	−0.26 (0.09)	−0.44 to −0.08		
Model 2: Age			0.53	.47
Intercept	0.25 (0.44)	−0.61 to 1.12		
Participants’ average age	0.01 (0.01)	−0.02 to 0.04		
Model 3: Type of interaction (reference: interacting with designed CAs^a^)			0.19	.66
Intercept	0.55 (0.20)	0.15 to 0.95		
Wizard of OZ	−0.17 (0.38)	−0.92 to 0.58		
Model 4: Health topic (reference: mental health)			1.57	.46
Intercept	0.60 (0.18)	0.24 to 0.96		
Promote health behavior	−0.59 (0.54)	−1.64 to 0.47		
Provide medical information and advice	−0.45 (0.63)	−1.69 to 0.78		
Model 5: Publication outlet (reference: journal publication)			3.39	.18
Intercept	0.32 (0.21)	−0.09 to 0.73		
Conference proceeding	0.74 (0.41)	−0.06 to 1.53		
Thesis and dissertation	0.07 (0.43)	−0.76 to 0.90		

a CAs: conversational agents.

#### Risk of Bias

Risk of bias was assessed for studies included in the meta-analysis. Regarding the overall assessment, one study was assessed to have a low risk of bias, 13 studies were rated as having some concerns, and 11 studies were assessed to have a high risk of bias. Detailed assessments across 5 domains for each study are shown in Multimedia Appendix 4.

#### Publication Bias

We first tested whether the overall effect sizes varied across different publication outlets: journal publication, conference proceeding, and thesis and dissertation. As shown in Tables 3-5, omnibus tests indicated no significant differences in the overall effect sizes for the evaluation of CA (*QM*_2_=0.19; *P*=.91), use of CA (*QM*_2_=1.95; *P*=.38), and psychological outcomes (*QM*_2_=3.39; *P*=.18) across 3 publication outlets.

We further assessed publication bias using funnel plots and Egger regression test. Funnel plots visually indicate bias, with symmetry suggesting its absence and asymmetry—such as missing points in the center or corners—indicating potential bias [87]. Egger test provides a statistical evaluation of funnel plot asymmetry, with significant results indicating bias [87]. As these methods were developed for traditional meta-analyses, we followed procedures used in prior 3-level meta-analyses [57,88], creating a new dataset for each outcome type by randomly selecting 1 effect size per study to generate funnel plots and perform Egger tests.

Four funnel plots are provided in Multimedia Appendix 5. No substantial asymmetry was observed in those funnel plots. The results of Egger regression tests indicated no evidence of publication bias for the evaluation of CA (*t*_18_=0.35; *P*=.73), use of CA (*t*_9_=−0.02; *P*=.98), psychological outcomes (*t_9_*=2.17; *P*=.06), and health outcomes (*t*_1_=0.50; *P*=.70).

#### Sensitivity Analysis

The leave-one-out method, which removes one study at a time and recalculates the overall effect size, was conducted for sensitivity analysis. The overall effect sizes remained stable for the evaluation of CA (ranging from 0.39 to 0.48), use of CA (ranging from 0.07 to 0.14), and psychological outcomes (ranging from 0.37 to 0.54). However, the pooled effect size for health outcomes was not robust to the exclusion of studies, with values ranging from 0.00 to 0.44. Specifically, excluding Albers et al [83] increased the overall effect size to 0.44 (*P*=.001), while excluding Lee et al [70] reduced it to 0.00 (*P*=.996). The lack of robustness for this outcome is probably due to the small number of studies, with only 3 studies examining the relevant variables.

## Discussion

### Principal Findings

The systematic review of 31 experimental studies identified 11 major strategies to operationalize communication competence in CAs for health. The most commonly used approach was the provision of empathetic responses, which was implemented in more than half of the reviewed studies, followed by self-disclosure. Other frequently examined communication competencies included contingent conversations, small talk, social etiquette, and explanation. Humor and emotional expressiveness were used less frequently, while personalization, open-ended questions, and partnerships were the least common strategies.

The 3-level meta-analyses of 25 studies demonstrated the effectiveness of CAs’ communication competence on multiple outcomes. Specifically, health CAs’ communication competence has a small-to-medium positive effect on users’ evaluations of CAs and psychological outcomes. However, its effectiveness did not extend to the use of CA and health outcomes. The moderator analyses indicated a slight decrease in the effect size for psychological outcomes along with the publication year. In contrast, the effect sizes were stable across participants’ average age, type of interaction, and health topics.

### Comparison With Prior Work

#### Communication Competence in Health Care CA

Most of the identified strategies to enhance CAs’ communication competence reflected the desired communication skills proposed in interpersonal communication competence [13]. For example, the provision of empathetic responses echoes the empathy dimension of communication competence in the interpersonal context. The strategy of self-disclosure also resembles a favorable quality in interpersonal communication competence. Whether someone can express emotion or feelings through nonverbal behaviors or vocal modulation is deemed expressiveness, which seems applicable only in interpersonal settings. However, 2 research studies manipulated expressiveness by managing emojis and emotionally expressive texts, mimicking mediated communication experience.

Beyond direct parallels to interpersonal competence, this review also identified nuanced features unique to CA contexts. Social etiquette and small talk support interaction management by enabling agents to follow social norms and engage in casual conversation. Contingency and personalization reflect *altercentrism*—attentiveness to others—through features such as referencing prior conversations or tailoring responses to users [41]. Humor, often implemented via jokes, aims to foster a relaxed atmosphere and mirrors the interpersonal concept of *social relaxation*. Notably, 3 interpersonal dimensions—assertiveness, immediacy, and environmental control—are absent in current CA studies.

The other 3 operationalizations of communication competence—explanation, open-ended questions, and partnership—reflected the important communication skills discussed in previous research on patient-provider communication. The strategy of explanation usually involves providing explanations after giving a recommendation or diagnosis. In patient-provider communication, offering sufficient explanations is a key aspect of the provider’s communication competence to ensure effective information exchange [26]. Although rooted in clinical contexts, this strategy has been applied in broader CA functions such as symptom checking and health advising. Partnership-building [89] and the use of open-ended questions [33], although foundational in medical communication, remain underutilized in CA design. Further studies on CAs in health can continue to use explanations to enhance CA capability, as well as strengthen its ability for open-ended questions and partnership building.

#### Effectiveness of CA Communication Competence

The positive effect size on users’ evaluations of the CA aligns with the computers are social actors paradigm, which suggests that individuals mindlessly apply social rules from human-human interaction when interacting with nonhuman entities that exhibit enough social cues [90]. In interpersonal communication, communication competence has been found to be a crucial factor in increasing people’s liking of the conversational partner and fostering more positive evaluations of the interaction [91,92]. As communication competence functions as a key social cue [93], individuals apply a similar mental model that favors CAs using various communication competence strategies, with a more positive perception of CA capabilities [38,71], stronger liking toward CA [74], and trust in CA [70]. The magnitude of the effect size highlights the importance of integrating diverse communication competence strategies, such as self-disclosure, empathetic responses, message contingency, and small talk when designing health care CAs to improve users’ evaluation of these CAs.

In addition, our analysis showed that the positive effects of CA communication competence further extended to users’ psychological outcomes. Previous reviews on CAs’ influence have primarily focused on outcomes related to user experience and health behaviors [11,14,21]. However, this meta-analysis underscores psychological outcomes as a crucial area of impact that can be significantly improved by incorporating communication competence strategies. Consistent with findings in human-human communication that communication competence is associated with improved affect and reduced stress [91,94], individuals interacting with CAs that exhibit higher communication competence benefit from various psychological improvements, such as increased positive emotions [60], cognitive reappraisal [41], and reduced emotional distress [40]. The improved psychological outcomes provide some support for the effectiveness of existing CA-based health interventions for addressing mental health problems, such as reduced anxiety [32] and depression [95]. The integration of communication competence modules is likely a contributing factor to their effectiveness. Previous reviews suggested personalization and empathetic response as 2 facilitators of intervention effectiveness [9]. Our findings further extend this effect to the broader category of CAs’ communication competence. Furthermore, as psychological outcomes function as key pathways linking interactions with CA to health behaviors and well-being, the small-to-medium effect size observed in our meta-analysis suggests the critical role of CA communication competence in various health contexts. Future health interventions can prioritize enhancing CA communication competence when designing CAs to optimize the efficacy.

The digital divide across age groups has long been observed and discussed [96,97]. Previous evidence suggested that younger generations tend to be more welcome to AI than older generations [46,47]. However, opposite to our expectation, our synthesized results indicate that users’ age does not influence the strength of the effects of CAs’ communication competence. Two thoughts arise from this observation. First, the meta-analysis used the average sample ages from the included studies as the analysis unit, consisting of no information about age distribution within each study. For example, an average age of 30 years could result from a sample age ranging from 18 to 60 years or from 20 to 40 years. Consequently, the moderator of average participants’ age does not fully capture the characteristics of the sample age. Second, this suggests that the limited knowledge of AI, reduced experience in using AI, and less positive attitudes toward AI among older adults may not be adequate to result in a significant difference in CA outcomes.

In addition to age, our analysis found that the type of interaction and the health topics did not significantly change the effect sizes for the evaluations of CAs and psychological outcomes. This finding alleviates the methodological and design concerns regarding whether certain interaction types are more appropriate for the experiment or whether CAs’ communication competence is more effective for specific health services than others. Considering the nonsignificant moderation effects of the interaction type, future studies on the communication competence of health care CAs can start from using prerecorded materials or the Wizard of Oz method in the pilot study to assess feasibility and refine study design before investing more time and resources in developing a CA prototype for the main study. The nonsignificant results from the moderation analyses regarding health topics underscore the broad generalizability and potential effectiveness of CAs’ communication competence across real-world health settings, including information gathering and distribution, automatic symptom diagnosis, and the provision of health advice.

The publication year was the only significant moderator that affected the effectiveness of CAs’ communication competence on the use of CA and psychological outcomes. Contrary to our expectations, the magnitude of the effect size slightly decreased in more recent publication years. One possible explanation for this diminishing effect is the decline in novelty effects. Users may initially enjoy interacting with CAs for the novelty of the implemented communication competence. However, this novelty effect fades gradually as more CAs incorporate relevant strategies. Another possibility is that as users get more familiar with CAs, they develop higher demands that current CAs’ communication competence cannot meet. Although this finding suggests a temporal change in the effect size, it should be interpreted with caution. The range of the publication year of the included studies is relatively narrow, with all studies examining psychological outcomes published after 2019. Therefore, it is uncertain to what extent this trend can be generalized to a longer period beyond the examined time frame.

### Limitations

Despite its intellectual merits, this paper has a few limitations. First, only 3 out of the 25 independent studies were included in the meta-analysis for health outcomes, which restricts our ability to draw robust conclusions on whether CAs’ enhanced communication competence leads to meaningful health improvements. As indicated by the large variance observed in the leave-one-out analysis, the pooled effect size on health outcomes needs to be interpreted with caution. In addition, the small number of studies limits our ability to conduct moderator analyses to explore primary factors contributing to the effect size heterogeneity. Therefore, the findings on health outcomes remain preliminary and need further research.

Second, despite the overall positive effects of CAs’ enhanced communication competence, it remains unclear which specific component drives these effects. Our findings indicate that researchers have implemented various strategies to enhance CAs’ communication competence. However, the limited research efforts on some strategies (eg, humor and personalization) constrain our ability to conduct meaningful subgroup analysis to compare the effectiveness of different strategies. In addition, each communication strategy can be operationalized in various ways across studies. For example, empathetic responses may involve expressions of sympathy, emotional validation, or comfort, and some studies implemented multiple components simultaneously [39,74,75]. This variation makes it challenging to isolate and assess which component contributes more to the effectiveness.

Third, given the nonsignificant moderation effects, heterogeneity in effect sizes may stem from unreported user attributes and interaction characteristics, such as prior CA experience and interaction duration. These factors likely influence users’ perceptions of communication competence but were largely absent in the included studies, limiting our ability to assess their impact. In addition, with only 1 study examining voice-based CAs, we could not compare them with text-based systems to examine whether the effects of communication competence vary across interaction modality. As voice-based CAs become more prevalent, their communication competence and effectiveness warrant further investigation.

### Future Directions

This review highlights several important directions for future research. First, the relatively limited focus on health outcomes underscores the need to move beyond user evaluations and examine whether enhanced communication competence of health CAs leads to tangible health improvement. Although health CAs are increasingly deployed in diverse health contexts, including treatment support, disease management, and physical health promotion [6,98], empirical evidence on how enhanced communication competence translates into clinical improvement remains limited. To better understand the practical and clinical values of CAs’ communication competence, future research can prioritize assessing its direct effects on various downstream health outcomes, such as health behavior change, treatment adherence, and symptom improvement.

Second, future research can delve deeper into different aspects of communication competence to better understand their effectiveness. As most of the included studies have focused on empathetic responses, future studies are encouraged to explore those understudied strategies and emerging strategies derived from interpersonal and patient-provider communication theories. As more empirical evidence accumulates, future meta-analyses can further disentangle different approaches of operationalization and compare their effectiveness to determine which aspects play more critical roles in health contexts.

Third, future research can expand the assessment of CAs’ communication competence in broader contexts and populations. The rapid development of LLMs presents emerging opportunities to enhance CAs’ communication competence. It is worth examining whether and how the integration of LLMs alters the effectiveness of CAs’ communication competence. In addition, as most included studies focused on general adult populations, future research can explore the effects among different age groups, particularly among children and older adults, to better understand the nuanced effects and inform the development of health interventions tailored to different ages.

In addition, future research can explore how enhanced CAs’ communication competence functions within complex clinical environments. Despite the significant effects of CAs’ communication competence on user-centered outcomes, few included studies examined how such enhanced competence integrates with real-world health care systems. In practice, health CAs are usually deployed as components within broader health care systems, working in collaboration with health care providers. Future studies can design empirical studies to investigate how CAs with enhanced communication competence influence health care systems and provider workflows, such as provider workload, system efficiency, and clinical assessment quality. Furthermore, researchers can examine the impact of embedding such CAs into clinical settings on patient-provider interactions, particularly patient expectations and compatibility with time-constrained encounters.

Finally, given the demonstrated effectiveness of CAs’ communication competence, future research can further advance the development of algorithms that equip CAs with different communication strategies. For example, one particular promising direction is enhancing CAs’ provision of empathetic responses. Recent research in AI systems has been devoting growing efforts to advance techniques that can detect and respond to users’ psychological states [99], which lays the critical technical foundation for implementing empathetic responses in CAs based on users’ current emotional states. Future studies can extend this line of work to implement primary communication competence strategies for health CAs across diverse health care contexts.

### Conclusions

This review summarized the operationalization of communication competence in CAs for health and evaluated its impacts on users’ evaluations of CAs, use of CA, psychological outcomes, and health outcomes. Through a systematic review, we identified 11 major strategies to build communication competence in CAs, including empathetic response, contingency, humor, small talk, emotional expressiveness, self-disclosure, personalization, social etiquette, explanation, open-ended questions, and partnership. The meta-analyses indicate that health CAs’ communication competence significantly improves users’ evaluations of CA and psychological outcomes. Furthermore, moderation analyses suggest that these positive effects are stable across different experiment methodologies and health contexts.

## Supplementary material

10.2196/76296 Multimedia Appendix 1 Complete list of literature search queries for each database.

10.2196/76296 Multimedia Appendix 2 Formulas used to convert effect sizes.

10.2196/76296 Multimedia Appendix 3 Key information extracted from each included study.

10.2196/76296 Multimedia Appendix 4 Plots for risk of bias assessments.

10.2196/76296 Multimedia Appendix 5 Funnel plots of 4 outcome variables.

10.2196/76296 Checklist 1 PRISMA checklist.

## Abbreviations

AI: artificial intelligence
CA: conversational agent
LLM: large language model
PRISMA: Preferred Reporting Items for Systematic Reviews and Meta-Analyses
RQ: research question
