# Supplementary material for: Effectiveness of Communication Competence in AI Conversational Agents for Health: Systematic Review and Meta-Analysis
Source: J Med Internet Res. 2025 Nov 3;27:e76296. doi: 10.2196/76296 (PMC12582511; doi:10.2196/76296)
Supplement: Multimedia Appendix 1 [file jmir-v27-e76296-s001.docx]

**Multimedia Appendix 1**

This appendix provides the complete list of search terms and search settings in each database.

**APA PsycInfo**

Search Area: Title, Abstract

Limiters - Publication Date: 2003/01/01-2023/12/31; Language: English; Document Type: Dissertation, Journal Article

**S1** = TI ( “conversational agent*” OR “communicative agent*” OR chatbot* OR “chat bot*” OR “artificial intelligence chatbot*” OR “AI chatbot*” OR chatterbot OR “intelligent agent*” OR “artificial agent*” OR “AI agent*” OR “conversational system*” OR “conversational AI” OR “conversational assistant*” OR “conversational bot*” OR “conversational interface” OR “dialog* system*” OR “dialogue agent*” OR “assistance technolog*” OR “digital assistant*” OR “virtual agent*” OR “relational agent*” OR “interactive agent*” OR “virtual assistant*” OR “virtual coach*” OR “virtual advisor*” OR “interface agent*” OR “virtual therapist*” ) AND TI ( “communicati* competenc*” OR “conversational skill*” OR “communication skill*” OR “conversational capabilit*” OR “communication capabilit*” OR “social skill*” OR emotion* OR socioemotion* OR emotive OR expression OR expressiv* OR “emoji” OR empath* OR understanding OR “small talk” OR “social cue*” OR “social signal” OR greeting* OR disclos* OR self-disclos* OR reciprocity OR personaliz* OR customiz* OR contingen* OR adaptab* OR interactiv* OR accommodation OR match* OR mimic* OR “linguistic style*” OR “language style*” OR “conversational style*” OR “chatting style*” OR joke* OR humor* OR polite* OR respect OR comfort OR “active listening” OR confidence OR immediacy ) AND TI ( experiment* OR “randomized controlled trial” OR “random* assign*” OR randomization OR quasi-experiment* OR “factorial design” )

**S2** = AB ( “conversational agent*” OR “communicative agent*” OR chatbot* OR “chat bot*” OR “artificial intelligence chatbot*” OR “AI chatbot*” OR chatterbot OR “intelligent agent*” OR “artificial agent*” OR “AI agent*” OR “conversational system*” OR “conversational AI” OR “conversational assistant*” OR “conversational bot*” OR “conversational interface” OR “dialog* system*” OR “dialogue agent*” OR “assistance technolog*” OR “digital assistant*” OR “virtual agent*” OR “relational agent*” OR “interactive agent*” OR “virtual assistant*” OR “virtual coach*” OR “virtual advisor*” OR “interface agent*” OR “virtual therapist*” ) AND AB ( “communicati* competenc*” OR “conversational skill*” OR “communication skill*” OR “conversational capabilit*” OR “communication capabilit*” OR “social skill*” OR emotion* OR socioemotion* OR emotive OR expression OR expressiv* OR “emoji” OR empath* OR understanding OR “small talk” OR “social cue*” OR “social signal” OR greeting* OR disclos* OR self-disclos* OR reciprocity OR personaliz* OR customiz* OR contingen* OR adaptab* OR interactiv* OR accommodation OR match* OR mimic* OR “linguistic style*” OR “language style*” OR “conversational style*” OR “chatting style*” OR joke* OR humor* OR polite* OR respect OR comfort OR “active listening” OR confidence OR immediacy ) AND AB ( experiment* OR “randomized controlled trial” OR “random* assign*” OR randomization OR quasi-experiment* OR “factorial design” )

**S1** OR **S2**

**Communication & Mass Media Complete**

Search Area: Title, Abstract

Limiters - Published Date: 2003/01/01-2023/12/31; Publication Type: Academic Journal, Conference Proceeding; Language: English

**S1** = TI ( “conversational agent*” OR “communicative agent*” OR chatbot* OR “chat bot*” OR “artificial intelligence chatbot*” OR “AI chatbot*” OR chatterbot OR “intelligent agent*” OR “artificial agent*” OR “AI agent*” OR “conversational system*” OR “conversational AI” OR “conversational assistant*” OR “conversational bot*” OR “conversational interface” OR “dialog* system*” OR “dialogue agent*” OR “assistance technolog*” OR “digital assistant*” OR “virtual agent*” OR “relational agent*” OR “interactive agent*” OR “virtual assistant*” OR “virtual coach*” OR “virtual advisor*” OR “interface agent*” OR “virtual therapist*” ) AND TI ( “communicati* competenc*” OR “conversational skill*” OR “communication skill*” OR “conversational capabilit*” OR “communication capabilit*” OR “social skill*” OR emotion* OR socioemotion* OR emotive OR expression OR expressiv* OR “emoji” OR empath* OR understanding OR “small talk” OR “social cue*” OR “social signal” OR greeting* OR disclos* OR self-disclos* OR reciprocity OR personaliz* OR customiz* OR contingen* OR adaptab* OR interactiv* OR accommodation OR match* OR mimic* OR “linguistic style*” OR “language style*” OR “conversational style*” OR “chatting style*” OR joke* OR humor* OR polite* OR respect OR comfort OR “active listening” OR confidence OR immediacy ) AND TI ( experiment* OR “randomized controlled trial” OR “random* assign*” OR randomization OR quasi-experiment* OR “factorial design” )

**S2** = AB ( “conversational agent*” OR “communicative agent*” OR chatbot* OR “chat bot*” OR “artificial intelligence chatbot*” OR “AI chatbot*” OR chatterbot OR “intelligent agent*” OR “artificial agent*” OR “AI agent*” OR “conversational system*” OR “conversational AI” OR “conversational assistant*” OR “conversational bot*” OR “conversational interface” OR “dialog* system*” OR “dialogue agent*” OR “assistance technolog*” OR “digital assistant*” OR “virtual agent*” OR “relational agent*” OR “interactive agent*” OR “virtual assistant*” OR “virtual coach*” OR “virtual advisor*” OR “interface agent*” OR “virtual therapist*” ) AND AB ( “communicati* competenc*” OR “conversational skill*” OR “communication skill*” OR “conversational capabilit*” OR “communication capabilit*” OR “social skill*” OR emotion* OR socioemotion* OR emotive OR expression OR expressiv* OR “emoji” OR empath* OR understanding OR “small talk” OR “social cue*” OR “social signal” OR greeting* OR disclos* OR self-disclos* OR reciprocity OR personaliz* OR customiz* OR contingen* OR adaptab* OR interactiv* OR accommodation OR match* OR mimic* OR “linguistic style*” OR “language style*” OR “conversational style*” OR “chatting style*” OR joke* OR humor* OR polite* OR respect OR comfort OR “active listening” OR confidence OR immediacy ) AND AB ( experiment* OR “randomized controlled trial” OR “random* assign*” OR randomization OR quasi-experiment* OR “factorial design” )

S1 OR S2

**ACM Digital Library**

Search Area: ACM Full-Text Collection

**S1** = "query": {Abstract:("conversational agent*" OR "communicative agent*" OR chatbot* OR "chat bot*" OR "artificial intelligence chatbot*" OR "AI chatbot*" OR chatterbot OR "intelligent agent*" OR "artificial agent*" OR "AI agent*" OR "conversational system*" OR "conversational AI" OR "conversational assistant*" OR "conversational bot*" OR "conversational interface" OR "dialog* system*" OR "dialogue agent*" OR "assistance technolog*" OR "digital assistant*" OR "virtual agent*" OR "relational agent*" OR "interactive agent*" OR "virtual assistant*" OR "virtual coach*" OR "virtual advisor*" OR "interface agent*" OR "virtual therapist*") AND Abstract:("communicati* competenc*" OR "conversational skill*" OR "communication skill*" OR "conversational capabilit*" OR "communication capabilit*" OR "social skill*" OR emotion* OR socioemotion* OR emotive OR expression OR expressiv* OR "emoji" OR empath* OR understanding OR "small talk" OR "social cue*" OR "social signal" OR greeting* OR disclos* OR self-disclos* OR reciprocity OR personaliz* OR customiz* OR contingen* OR adaptab* OR interactiv* OR accommodation OR match* OR mimic* OR "linguistic style*" OR "language style*" OR "conversational style*" OR "chatting style*" OR joke* OR humor* OR polite* OR respect OR comfort OR "active listening" OR confidence OR immediacy) AND Abstract:(experiment* OR "randomized controlled trial" OR "random* assign*" OR randomization OR quasi-experiment* OR "factorial design")} "filter": {E-Publication Date: (01/01/2003 TO 12/31/2023)},{ACM Content: DL}

**S2** = "query": { Title:("conversational agent*" OR "communicative agent*" OR chatbot* OR "chat bot*" OR "artificial intelligence chatbot*" OR "AI chatbot*" OR chatterbot OR "intelligent agent*" OR "artificial agent*" OR "AI agent*" OR "conversational system*" OR "conversational AI" OR "conversational assistant*" OR "conversational bot*" OR "conversational interface" OR "dialog* system*" OR "dialogue agent*" OR "assistance technolog*" OR "digital assistant*" OR "virtual agent*" OR "relational agent*" OR "interactive agent*" OR "virtual assistant*" OR "virtual coach*" OR "virtual advisor*" OR "interface agent*" OR "virtual therapist*") AND Title:("communicati* competenc*" OR "conversational skill*" OR "communication skill*" OR "conversational capabilit*" OR "communication capabilit*" OR "social skill*" OR emotion* OR socioemotion* OR emotive OR expression OR expressiv* OR "emoji" OR empath* OR understanding OR "small talk" OR "social cue*" OR "social signal" OR greeting* OR disclos* OR self-disclos* OR reciprocity OR personaliz* OR customiz* OR contingen* OR adaptab* OR interactiv* OR accommodation OR match* OR mimic* OR "linguistic style*" OR "language style*" OR "conversational style*" OR "chatting style*" OR joke* OR humor* OR polite* OR respect OR comfort OR "active listening" OR confidence OR immediacy) AND Title:(experiment* OR "randomized controlled trial" OR "random* assign*" OR randomization OR quasi-experiment* OR "factorial design") } "filter": { E-Publication Date: (01/01/2003 TO 12/31/2023), ACM Content: DL }

S1 OR S2

**Web of Science Core Collection**

Search Area: Topic (including title, abstract, author keywords, and Keywords Plus)

TS=(“conversational agent*” OR “communicative agent*” OR chatbot* OR “chat bot*” OR “artificial intelligence chatbot*” OR “AI chatbot*” OR chatterbot OR “intelligent agent*” OR “artificial agent*” OR “AI agent*” OR “conversational system*” OR “conversational AI” OR “conversational assistant*” OR “conversational bot*” OR “conversational interface” OR “dialog* system*” OR “dialogue agent*” OR “assistance technolog*” OR “digital assistant*” OR “virtual agent*” OR “relational agent*” OR “interactive agent*” OR “virtual assistant*” OR “virtual coach*” OR “virtual advisor*” OR “interface agent*” OR “virtual therapist*”) AND TS=(“communicati* competenc*” OR “conversational skill*” OR “communication skill*” OR “conversational capabilit*” OR “communication capabilit*” OR “social skill*” OR emotion* OR socioemotion* OR emotive OR expression OR expressiv* OR “emoji” OR empath* OR understanding OR “small talk” OR “social cue*” OR “social signal” OR greeting* OR disclos* OR self-disclos* OR reciprocity OR personaliz* OR customiz* OR contingen* OR adaptab* OR interactiv* OR accommodation OR match* OR mimic* OR “linguistic style*” OR “language style*” OR “conversational style*” OR “chatting style*” OR joke* OR humor* OR polite* OR respect OR comfort OR “active listening” OR confidence OR immediacy) AND TS=(experiment* OR “randomized controlled trial” OR “random* assign*” OR randomization OR quasi-experiment* OR “factorial design”) AND PY=(2003-2023) AND DT=(Article OR Proceedings Paper) AND LA=(English)

**Scopus**

Search Area: Title, abstract, keywords

( TITLE-ABS-KEY ( “conversational agent*” OR “communicative agent*” OR chatbot* OR “chat bot*” OR “artificial intelligence chatbot*” OR “AI chatbot*” OR chatterbot OR “intelligent agent*” OR “artificial agent*” OR “AI agent*” OR “conversational system*” OR “conversational AI” OR “conversational assistant*” OR “conversational bot*” OR “conversational interface” OR “dialog* system*” OR “dialogue agent*” OR “assistance technolog*” OR “digital assistant*” OR “virtual agent*” OR “relational agent*” OR “interactive agent*” OR “virtual assistant*” OR “virtual coach*” OR “virtual advisor*” OR “interface agent*” OR “virtual therapist*” ) AND TITLE-ABS-KEY ( “communicati* competenc*” OR “conversational skill*” OR “communication skill*” OR “conversational capabilit*” OR “communication capabilit*” OR “social skill*” OR emotion* OR socioemotion* OR emotive OR expression OR expressiv* OR “emoji” OR empath* OR understanding OR “small talk” OR “social cue*” OR “social signal” OR greeting* OR disclos* OR self-disclos* OR reciprocity OR personaliz* OR customiz* OR contingen* OR adaptab* OR interactiv* OR accommodation OR match* OR mimic* OR “linguistic style*” OR “language style*” OR “conversational style*” OR “chatting style*” OR joke* OR humor* OR polite* OR respect OR comfort OR “active listening” OR confidence OR immediacy ) AND TITLE-ABS-KEY ( experiment* OR “randomized controlled trial” OR “random* assign*” OR randomization OR quasi-experiment* OR “factorial design” ) ) AND LANGUAGE ( english ) AND SRCTYPE ( p OR j ) AND PUBYEAR > 2003 AND PUBYEAR < 2024

**ProQuest Dissertations & Theses**

Search Area: Title, abstract

Additional limits - Date: From January 01 2003 to December 31 2023; Language: English

**S1**=title("conversational agent*" OR "communicative agent*" OR chatbot* OR "chat bot*" OR "artificial intelligence chatbot*" OR "AI chatbot*" OR chatterbot OR "intelligent agent*" OR "artificial agent*" OR "AI agent*" OR "conversational system*" OR "conversational AI" OR "conversational assistant*" OR "conversational bot*" OR "conversational interface" OR "dialog* system*" OR "dialogue agent*" OR "assistance technolog*" OR "digital assistant*" OR "virtual agent*" OR "relational agent*" OR "interactive agent*" OR "virtual assistant*" OR "virtual coach*" OR "virtual advisor*" OR "interface agent*" OR "virtual therapist*") AND title("communicati* competenc*" OR "conversational skill*" OR "communication skill*" OR "conversational capabilit*" OR "communication capabilit*" OR "social skill*" OR emotion* OR socioemotion* OR emotive OR expression OR expressiv* OR "emoji" OR empath* OR understanding OR "small talk" OR "social cue*" OR "social signal" OR greeting* OR disclos* OR self-disclos* OR reciprocity OR personaliz* OR customiz* OR contingen* OR adaptab* OR interactiv* OR accommodation OR match* OR mimic* OR "linguistic style*" OR "language style*" OR "conversational style*" OR "chatting style*" OR joke* OR humor* OR polite* OR respect OR comfort OR "active listening" OR confidence OR immediacy) AND title(experiment* OR "randomized controlled trial" OR "random* assign*" OR randomization OR quasi-experiment* OR "factorial design")

**S2** = abstract("conversational agent*" OR "communicative agent*" OR chatbot* OR "chat bot*" OR "artificial intelligence chatbot*" OR "AI chatbot*" OR chatterbot OR "intelligent agent*" OR "artificial agent*" OR "AI agent*" OR "conversational system*" OR "conversational AI" OR "conversational assistant*" OR "conversational bot*" OR "conversational interface" OR "dialog* system*" OR "dialogue agent*" OR "assistance technolog*" OR "digital assistant*" OR "virtual agent*" OR "relational agent*" OR "interactive agent*" OR "virtual assistant*" OR "virtual coach*" OR "virtual advisor*" OR "interface agent*" OR "virtual therapist*") AND abstract("communicati* competenc*" OR "conversational skill*" OR "communication skill*" OR "conversational capabilit*" OR "communication capabilit*" OR "social skill*" OR emotion* OR socioemotion* OR emotive OR expression OR expressiv* OR "emoji" OR empath* OR understanding OR "small talk" OR "social cue*" OR "social signal" OR greeting* OR disclos* OR self-disclos* OR reciprocity OR personaliz* OR customiz* OR contingen* OR adaptab* OR interactiv* OR accommodation OR match* OR mimic* OR "linguistic style*" OR "language style*" OR "conversational style*" OR "chatting style*" OR joke* OR humor* OR polite* OR respect OR comfort OR "active listening" OR confidence OR immediacy) AND abstract(experiment* OR "randomized controlled trial" OR "random* assign*" OR randomization OR quasi-experiment* OR "factorial design")

[**S1**] OR [**S2**]

**PubMed**

Search Area: Title, abstract

Limiters - Publication Year: 2003/01/01 – 2023/12/31; Language: English

(("conversational agent*"[Title/Abstract] OR "communicative agent*"[Title/Abstract] OR "chatbot*"[Title/Abstract] OR "chat bot*"[Title/Abstract] OR "artificial intelligence chatbot*"[Title/Abstract] OR "ai chatbot*"[Title/Abstract] OR "chatterbot"[Title/Abstract] OR "intelligent agent*"[Title/Abstract] OR "artificial agent*"[Title/Abstract] OR "ai agent*"[Title/Abstract] OR "conversational system*"[Title/Abstract] OR "conversational AI"[Title/Abstract] OR "conversational assistant*"[Title/Abstract] OR "conversational bot*"[Title/Abstract] OR "conversational interface"[Title/Abstract] OR "dialog* system*"[Title/Abstract] OR "dialogue agent*"[Title/Abstract] OR "assistance technolog*"[Title/Abstract] OR "digital assistant*"[Title/Abstract] OR "virtual agent*"[Title/Abstract] OR "relational agent*"[Title/Abstract] OR "interactive agent*"[Title/Abstract] OR "virtual assistant*"[Title/Abstract] OR "virtual coach*"[Title/Abstract] OR "virtual advisor*"[Title/Abstract] OR "interface agent*"[Title/Abstract] OR "virtual therapist*"[Title/Abstract]) AND ("communicati* competenc*"[Title/Abstract] OR "conversational skill*"[Title/Abstract] OR "communication skill*"[Title/Abstract] OR "conversational capabilit*"[Title/Abstract] OR "communication capabilit*"[Title/Abstract] OR "social skill*"[Title/Abstract] OR "emotion*"[Title/Abstract] OR "socioemotion*"[Title/Abstract] OR "emotive"[Title/Abstract] OR "expression"[Title/Abstract] OR "expressiv*"[Title/Abstract] OR "emoji"[Title/Abstract] OR "empath*"[Title/Abstract] OR "understanding"[Title/Abstract] OR "small talk"[Title/Abstract] OR "social cue*"[Title/Abstract] OR "social signal"[Title/Abstract] OR "greeting*"[Title/Abstract] OR "disclos*"[Title/Abstract] OR "self disclos*"[Title/Abstract] OR "reciprocity"[Title/Abstract] OR "personaliz*"[Title/Abstract] OR "customiz*"[Title/Abstract] OR "contingen*"[Title/Abstract] OR "adaptab*"[Title/Abstract] OR "interactiv*"[Title/Abstract] OR "accommodation"[Title/Abstract] OR "match*"[Title/Abstract] OR "mimic*"[Title/Abstract] OR "linguistic style*"[Title/Abstract] OR "language style*"[Title/Abstract] OR "conversational style*"[Title/Abstract] OR "joke*"[Title/Abstract] OR "humor*"[Title/Abstract] OR "polite*"[Title/Abstract] OR "respect"[Title/Abstract] OR "comfort"[Title/Abstract] OR "active listening"[Title/Abstract] OR "confidence"[Title/Abstract] OR "immediacy"[Title/Abstract]) AND ("experiment*"[Title/Abstract] OR "randomized controlled trial"[Title/Abstract] OR "random* assign*"[Title/Abstract] OR "randomization"[Title/Abstract] OR "quasi experiment*"[Title/Abstract] OR "factorial design"[Title/Abstract]) AND 2003/01/01:2023/12/31[Date - Publication]) AND (english[Filter])
