# Supplementary material for: Effectiveness of Communication Competence in AI Conversational Agents for Health: Systematic Review and Meta-Analysis
Source: J Med Internet Res. 2025 Nov 3;27:e76296. doi: 10.2196/76296 (PMC12582511; doi:10.2196/76296)
Supplement: Multimedia Appendix 2 [file jmir-v27-e76296-s002.docx]

**Multimedia Appendix 2**

This appendix lists all the formulars we used to convert different statistics to Cohen *d* and Hedges *g*.

**Table S1. Formulars used to convert statistics to Hedges *g*.**

| Statistics | Convert Formular | Source |
| --- | --- | --- |
| Cohen *d* | $g=d\left( 1-\frac{3}{4df-1} \right)$ | Borenstein and Hedges (2019) [53] |
| t-test statistics (t) | $d= t\sqrt{\frac{n_{1}+n_{2}}{n_{1}n_{2}}}$ | Borenstein and Hedges (2019) [53] |
| One-way ANOVA F statistics (F) | $d= \pm\sqrt{\frac{F(n_{1}+n_{2})}{n_{1}n_{2}}}$ | Borenstein and Hedges (2019) [53] |
| Zero-order correlations (r) | $d= \frac{2r}{\sqrt{1-r^{2}}}$ | Cooper et al (2017) [52] |
| Odds ratio (OR) | $d=log(OR)\times\frac{\sqrt{3}}{\pi}$ | Borenstein et al (2009) [54] |
