# Supplementary material for: Effectiveness of Communication Competence in AI Conversational Agents for Health: Systematic Review and Meta-Analysis
Source: J Med Internet Res. 2025 Nov 3;27:e76296. doi: 10.2196/76296 (PMC12582511; doi:10.2196/76296)
Supplement: Multimedia Appendix 3 [file jmir-v27-e76296-s003.docx]

Multimedia Appendix 3.

Table S1. Summary of the key information from the studies included in the review.

| First author, publication year | Study design | Modality | Type of interaction | Health context | Specific functions | Outcomes: Evaluation of CA | Outcomes: Use of CA | Psychological outcomes | Health outcomes |
| --- | --- | --- | --- | --- | --- | --- | --- | --- | --- |
| Li et al (2023)^*^ [49] | Within subject | Text-based | Pre-recorded materials | AI-powered tele-triage | Collect information about the patient’s symptoms.  Inform patients of the transfer to the appropriate physician. | - Mindful anthropomorphism - Mindless anthropomorphism - Social presence - Privacy concern^†^ - Trust in CA^†^ - Satisfaction with CA^†^ | - Intention to disclose information^†^ - Intention to continuously use^†^ |  |  |
| Meng et al (2023)^*^ [41] | Between subject | Text-based | Wizard of Oz | Mental health | Engage in a supportive conversation to discuss a personal stressor. | - Perceived warmth - Perceived competence |  | - Cognitive reappraisal - Emotional validation - Reduced emotional distress^†^ |  |
| Liu et al (2022)^*^ [69] | Between subject | Text-based | Interact with designed CA | Provide medical information and advice | Ask patient information and symptoms.  Provide a symptom diagnosis.  Offer health advice. | - Perceived benefits of using chatbot - Privacy concerns | - Usage intention | - Self-efficacy in diagnosis and treatment |  |
| Trzebiński et al (2023)^*^ [73] | Between subject | Text-based | Interact with designed CA | Promote health behavior | Address user’s questions and concerns about the COVID-19 vaccines to support COVID-19 vaccination. | - Perceived user autonomy | - Chatbot patronage intention |  | - Vaccination intention shift^†^ |
| He et al (2022)^*^ [39] | Between subject | Text-based | Interact with designed CA | Promote health behavior | Conduct an intake interview for smoking cessation:  Asking standard intake questions.  Provide personalized feedback.  Discuss motivations for quitting smoking. | - Engagement with the chatbot - Therapeutic alliance - Perceived empathy - Perceived communication competence^†^ |  |  | - Motivation to quit smoking |
| Woodcock et al (2021)^*^ [65] | Between subject | Text-based | Pre-recorded materials | Provide medical information and advice | Collect information about symptoms.  Provides a symptom diagnosis.  Offer appointment booking in cases of serious symptoms. | - Faith in the symptom checker - Comprehension of provided explanation - Perceived depth of information from symptom checker |  |  |  |
| El Hefny et al (2021)^*^ [74] | Within subject | Text-based | Interact with designed CA | Provide medical information and advice | Provide COVID-19 information:  Track the number of cases per country.  Provide health advice.  Answer FAQs.  Offer symptoms checking. | - Likability of chatbot - Trustworthiness of chatbot |  |  |  |
| Pecune et al (2020) [42] | Between subject | Text-based | Interact with designed CA | Promote health behavior | Collect information about user’s eating habit and preferences.  Recommends healthy recipes accordingly. | - Perceived quality of the interaction - Perceived quality of the conversational recommender system |  |  | - Intention the cook the recommended recipes |
| Gotthardt et al (2022)^*^ [64] | Between subject | Voice-based | Interact with designed CA | Mental health | Ask the user about current emotional state.  Deliver cognitive behavioral therapy. | - Usability (SUS) - Satisfaction and acceptance (CSQ-I) - Perceived usability and acceptance (NPS) ^†^ |  |  |  |
| Ho (2018)^*^ [67] | Between subject | Text-based | Wizard of Oz | Mental health | Engages in a supportive conversation about the negative feelings of their cognitive task results. | - Perceived competence - Perceived objectivity | - Amount of emotional disclosure - Disclosure of negative emotions - Amount of questioning the partner | - Emotional relief - Negative affect - Perception of coping |  |
| Albers et al (2022)^*^ [83] | Mixed design | Text-based | Interact with designed CA | Promote health behavior | Send persuasive messages to encourage people to do preparatory activities for quitting smoking and increasing physical activity. |  |  | - Perceived motivational impact | - Efforts into assigned activities |
| Ghandeharioun (2019a)^*^ [60], (2019b)^*^ [61] | Between subject | Text-based | Interact with designed CA | Mental health | Track mood.  Deliver appropriate mental health interventions accordingly. |  | - Response latency - Frequency of response | - Positive emotions |  |
| Kobori et al (2016) [80] | Within subject | Text-based | Interact with designed CA | Promote health behavior | Track food and drink consumption for the previous day to obtain dietary records. | - Ratings of CA (i.e., response, meaningfulness, fun, naturalness, warmth, liveliness, simplicity) | - Intention to reuse |  |  |
| Meng and Dai (2021)^*^  [71] | Between subject | Text-based | Interact with designed CA | Mental health | Engage in a supportive conversation about a personal stressor. | - Perceived supportiveness of chatbot^†^ |  | - Stress reduction - Worry reduction |  |
| Lee et al (2020)^*^ [70] | Between subject | Text-based | Interact with designed CA | Mental health | Prompt daily journaling on emotional states and sensitive questions.  Share user’s self-disclosure to a mental health professional. | - Trust in chatbot | - Self-disclosure of information, thoughts, and feelings to chatbot | - Trust in mental health professional | - Data sharing behaviors - Disclosure of information, thoughts, and feelings to mental health professional |
| Liu and Sundar (2018)  Study 1^*^ [38] | Between subject | Text-based | Pre-recorded materials | Provide medical information and advice | Provide medical advice on sexually transmitted diseases based on the symptoms described by the user. | - Perceived chatbot sadness for user’s suffering - Perceived chatbot’s recognition of user feeling - Perceived chatbot’s understanding of user feeling - Chatbot message supportiveness - Chatbot message effectiveness - Sincerity in chatbot response^†^ - Spine-tingling perception of chatbot - Perceived eeriness - Belief in robotic feelings^†^ |  |  |  |
| Liu and Sundar (2018)  Study 2^*^ [38] | Between subject | Text-based | Interact with designed CA | Provide medical information and advice | Provide medical advice on sexually transmitted diseases based on the symptoms described by the user. | - All the variables measured in Study 1 - Perceived novelty of the interaction^†^ - Likeability of chatbot^†^ - Perceived intelligence of chatbot^†^ - Attitude towards the overall interaction^†^ - Belief in robotic intelligence^†^ |  | - Feeling of being supported^†^ |  |
| De Boni et al (2008)^*^  [79] | Mixed design | Text-based | Interact with designed CA | Promote health behavior | Collect information about barriers to exercise.  Provide a barrier-specific solution.  Confirm the appropriateness of the solution. | - Liking of the system (i.e., anonymity/avoid embarrassment^†^, convenience/quick response^†^, easy-to-use, getting advice/new ideas^†^, fun/novel^†^, like talking to a friend^†^) - Rating of the system (i.e., polite, professional, considerate, genuine, recognizing user, approachable) - Perception of appropriate level of system humor - Perception of enjoyable to use - Perception of being too impersonal - Perception of being too abrupt | - Intention to use system again |  |  |
| Kraus et al (2021)^*^ [75] | Between subject | Text-based | Interact with designed CA | Mental health | Conduct a daily mood check-in. | - Trust in chatbot - Reliability on chatbot - Competence of chatbot - Understandability of chatbot - Personal attachment to chatbot - Faith in chatbot - Usability of chatbot |  |  |  |
| Rains and High (2021) ^*^ [72] | Mixed design | Text-based | Interact with designed CA | Mental health | Engages in a supportive conversation to discuss a personal stressor. |  |  | - Reduction in emotional distress - Reappraisal - Validation |  |
| Mai et al (2021)^*^ [68] | Between subject | Text-based | Wizard of Oz | Mental health | Encourage students to describe their experiences with exam anxiety.  Discusses potential coping options to reduce or overcome exam anxiety. | - Perceived chatbot rapport - Rating of chatbot helpfulness | - Breadth of self-disclosure - Depth of self-disclosure |  |  |
| Mai et al (2022a)^*^ [81] | Between subject | Text-based | Interact with designed CA | Mental health | Encourage students to describe their experiences with exam anxiety.  Discusses potential coping options to reduce or overcome exam anxiety. | - Technical evaluation of chatbot^†^ - Perceived usability and acceptance of chatbot - Working alliance |  |  |  |
| You et al (2023)^*^ [77] | Within subject | Text-based | Interact with designed CA | Provide medical information and advice | Collect information about symptoms.  Provide a symptom diagnosis. | - Trust - Satisfaction - Efficiency - Likability - Usefulness - Transparency - Human likeness - Empathy - Usability (i.e., mental demand, performance, effort, frustration) |  |  |  |
| Beattie (2023)^*^ [66] | Between subject | Text-based | Wizard of Oz | Mental health | Engage in a supportive conversation about a personal stressor. | - Provider sensitivity | - Willingness for future interaction | - Affect improvement - Cognitive reappraisal |  |
| Rains et al (2020)^*^  (Study 1, Study 2) [40] | Between subject | Text-based | Interact with designed CA | Mental health | Engage in a supportive conversation to discuss a personal stressor. |  | - Number of positive emotion words - Number of negative emotion words - Number of cognitive mechanism words | - Change in emotional distress - Reappraisal - Perceived validation |  |
| Mai et al (2022b) [82] | Between subject | Text-based | Interact with designed CA | Mental health | Encourage students to describe their experiences with exam anxiety.  Discusses potential coping options to reduce or overcome exam anxiety. | - Perceived personality of chatbot - Satisfaction - Working alliance |  |  |  |
| Rains et al (2020)^*^ [76] | Between subject | Text-based | Interact with designed CA | Mental health | Engage in a supportive conversation to discuss a personal stressor. | - Evaluation of bot | - Number of words - Number of positive emotion words - Number of negative emotion words - Number of insight words | - Change in emotional distress |  |
| Lopatovska et al (2022a) [62], (2022b) [63] | Single group pre-post design | Voice-based | Interact with designed CA | Mental health | Provide strategies to distract negative feelings and reduce adolescents' loneliness. | - Rating of the interaction |  | - Loneliness - Positive affect - Negative affect - Perceived social support |  |
| Buzcu et al (2023) [84] | Within subject | Text-based | Interact with designed CA | Promote health behavior | Recommend food recipes to users based on users’ eating habits, allergies, and health needs. | - Sociability - Information completeness - System usability | - Intention to use the system |  |  |
| Lin et al (2023) [78] | Single group pre-post design | Text-based | Interact with designed CA | Mental health | Provide positive psychology content to improve individual mental health. | - System usability - Overall satisfaction - Content satisfaction - Perceived helpfulness, understanding, relevance |  | - Psychological well-being - Life satisfaction |  |

^a^ Studies marked with * were included in the meta-analysis.

^b^ Outcome variables marked with ^†^ were excluded from the meta-analysis because of insufficient information to calculate effect sizes.
