# Supplementary material for: Effectiveness of Communication Competence in AI Conversational Agents for Health: Systematic Review and Meta-Analysis
Source: J Med Internet Res. 2025 Nov 3;27:e76296. doi: 10.2196/76296 (PMC12582511; doi:10.2196/76296)

**Multimedia Appendix 4**

This multimedia appendix presents the plots for risk of bias assessment.

**Figure S1.** Traffic light plot for each individual study.

*
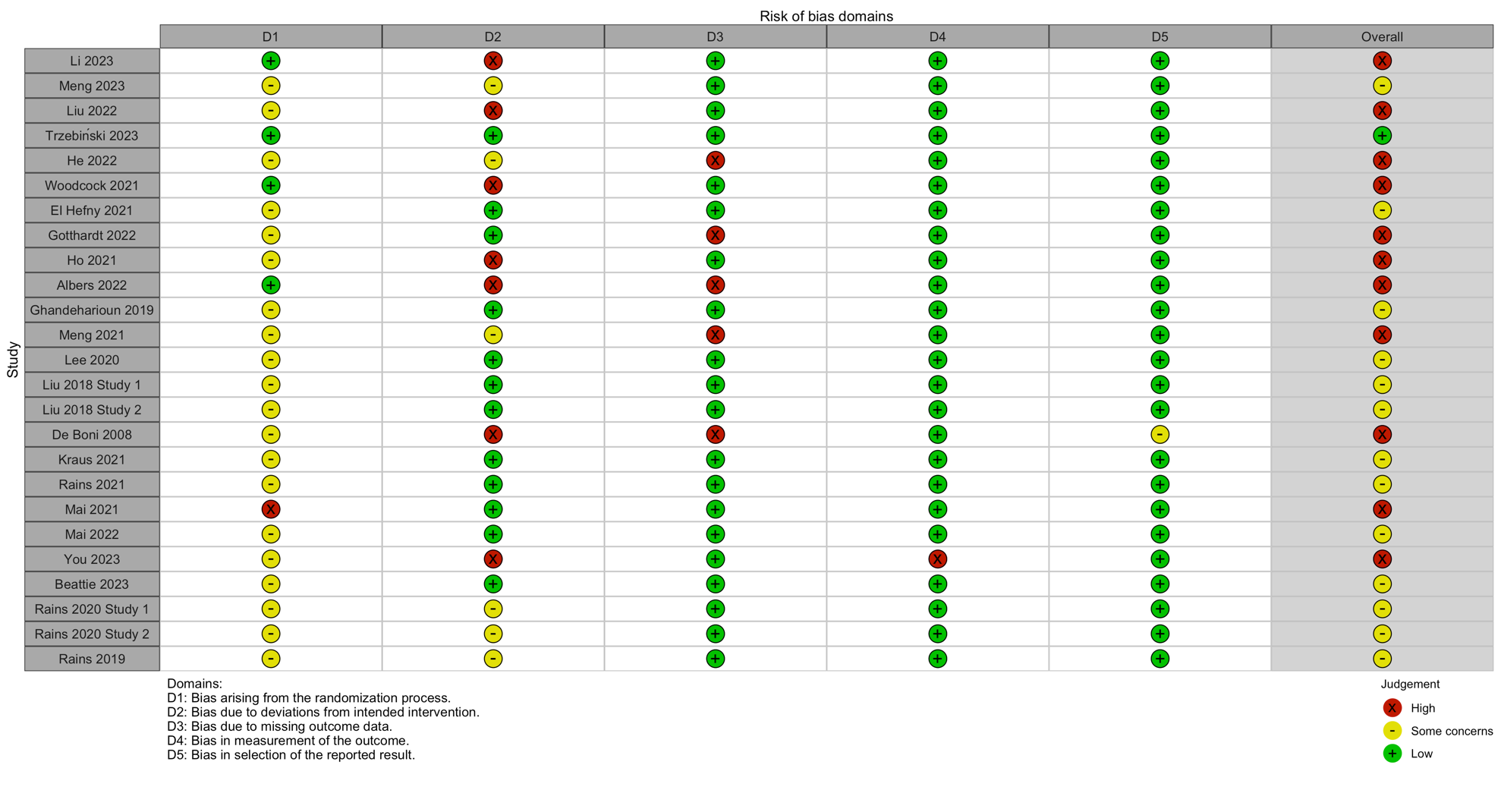
*

**Figure S2.** Bar plot of the distribution of risk of bias.


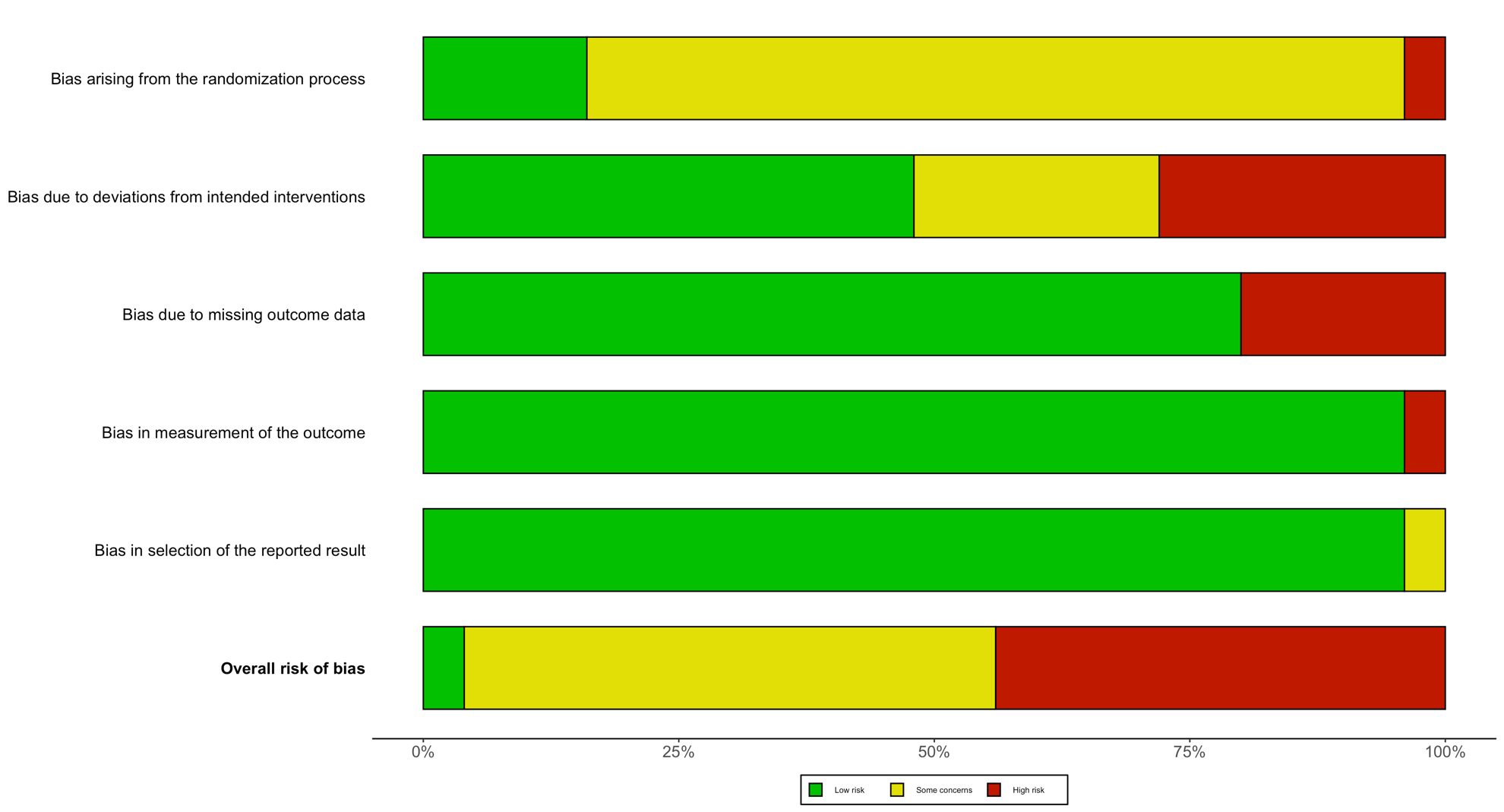

Supplement: Multimedia Appendix 4 [file jmir-v27-e76296-s004.docx]
