# Supplementary material for: Effectiveness of Communication Competence in AI Conversational Agents for Health: Systematic Review and Meta-Analysis
Source: J Med Internet Res. 2025 Nov 3;27:e76296. doi: 10.2196/76296 (PMC12582511; doi:10.2196/76296)

**Multimedia Appendix 5**

This appendix presents the funnel plots for all four outcomes variables.

**Figure S1.** Funnel plots for evaluation of CA.

*
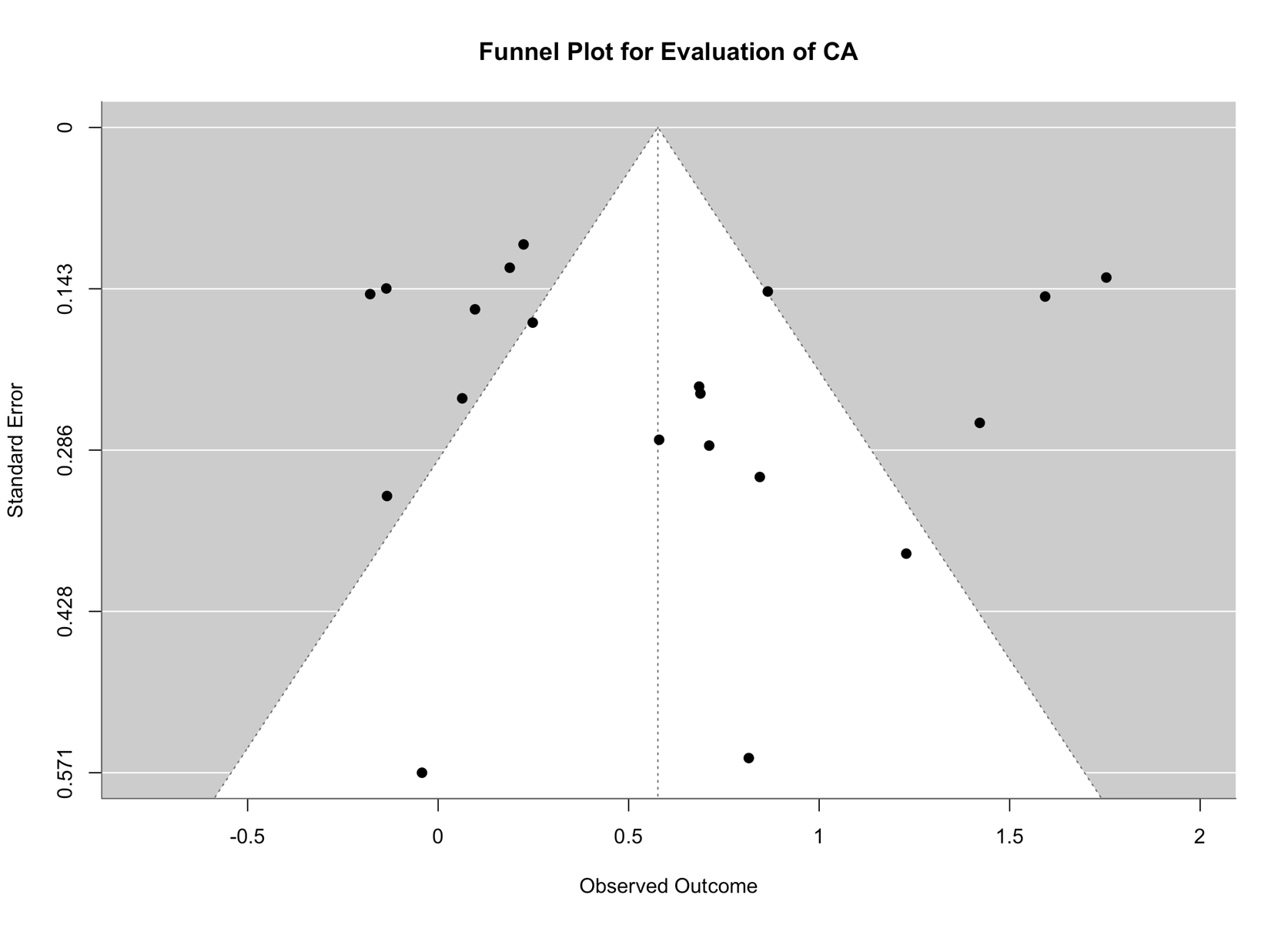
*

**Figure S2.** Funnel plots for use of CA.

*
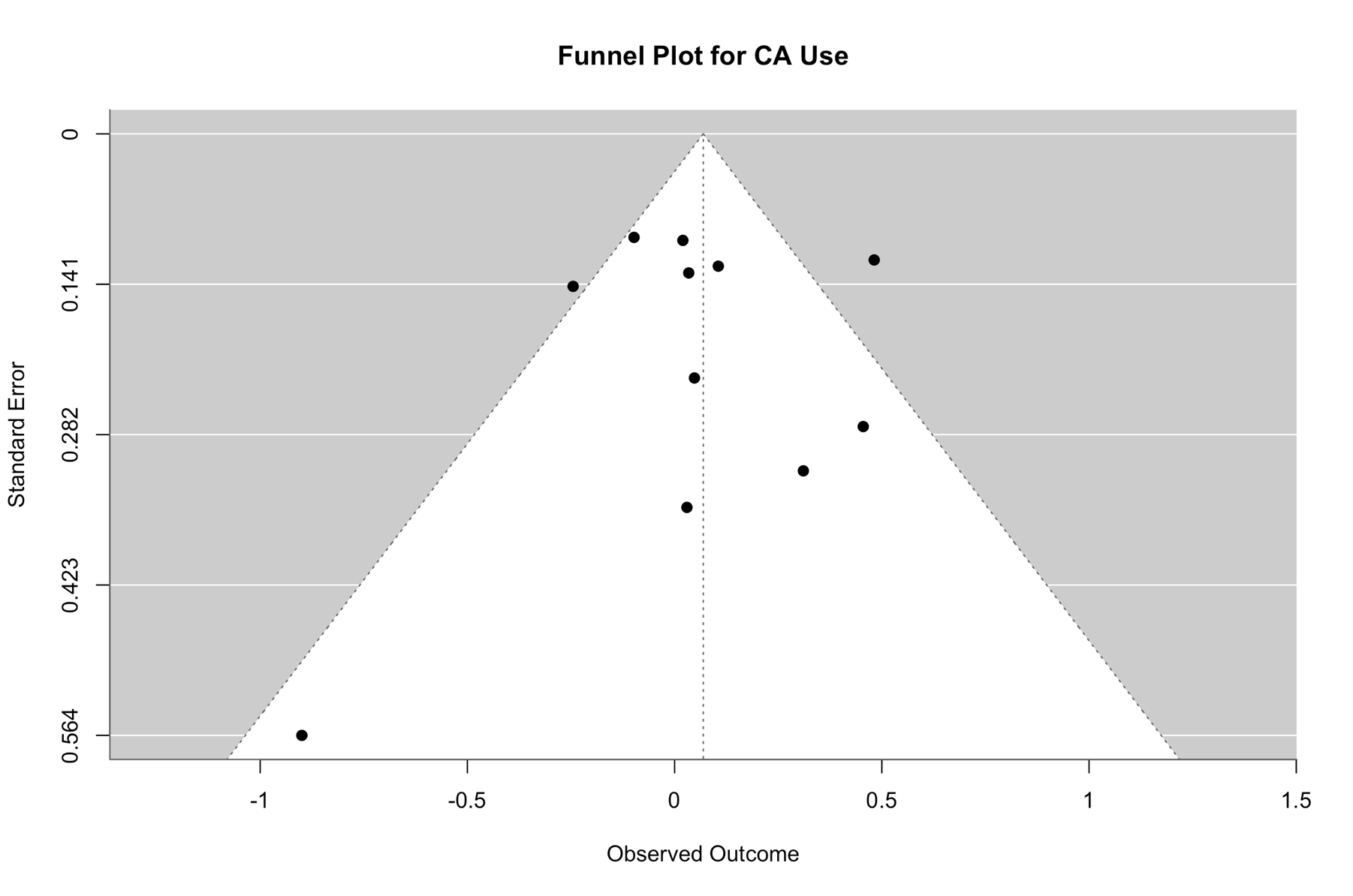
*

**Figure S3.** Funnel plots for psychological outcomes.

*
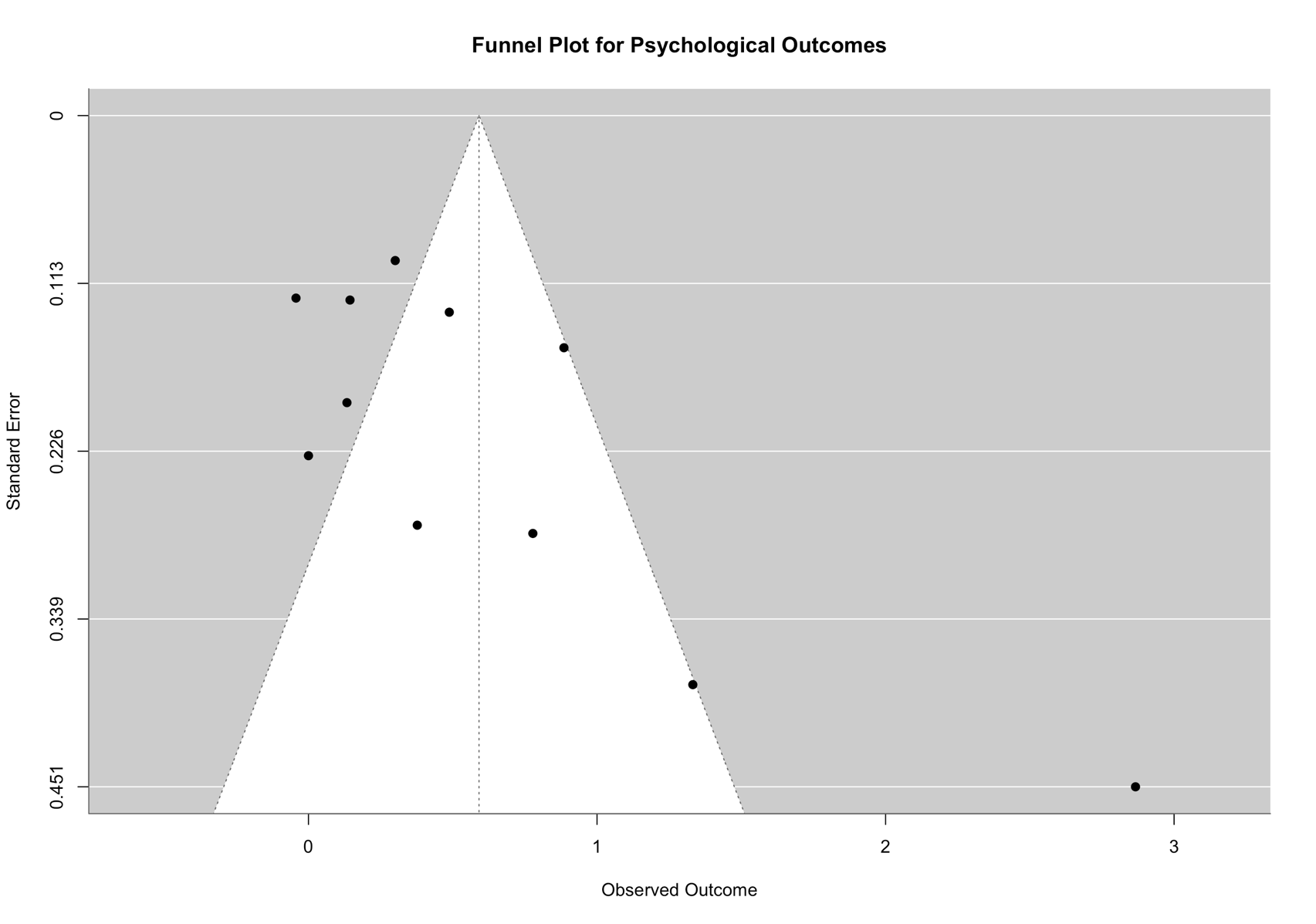
*

**Figure S4.** Funnel plots for health outcomes.


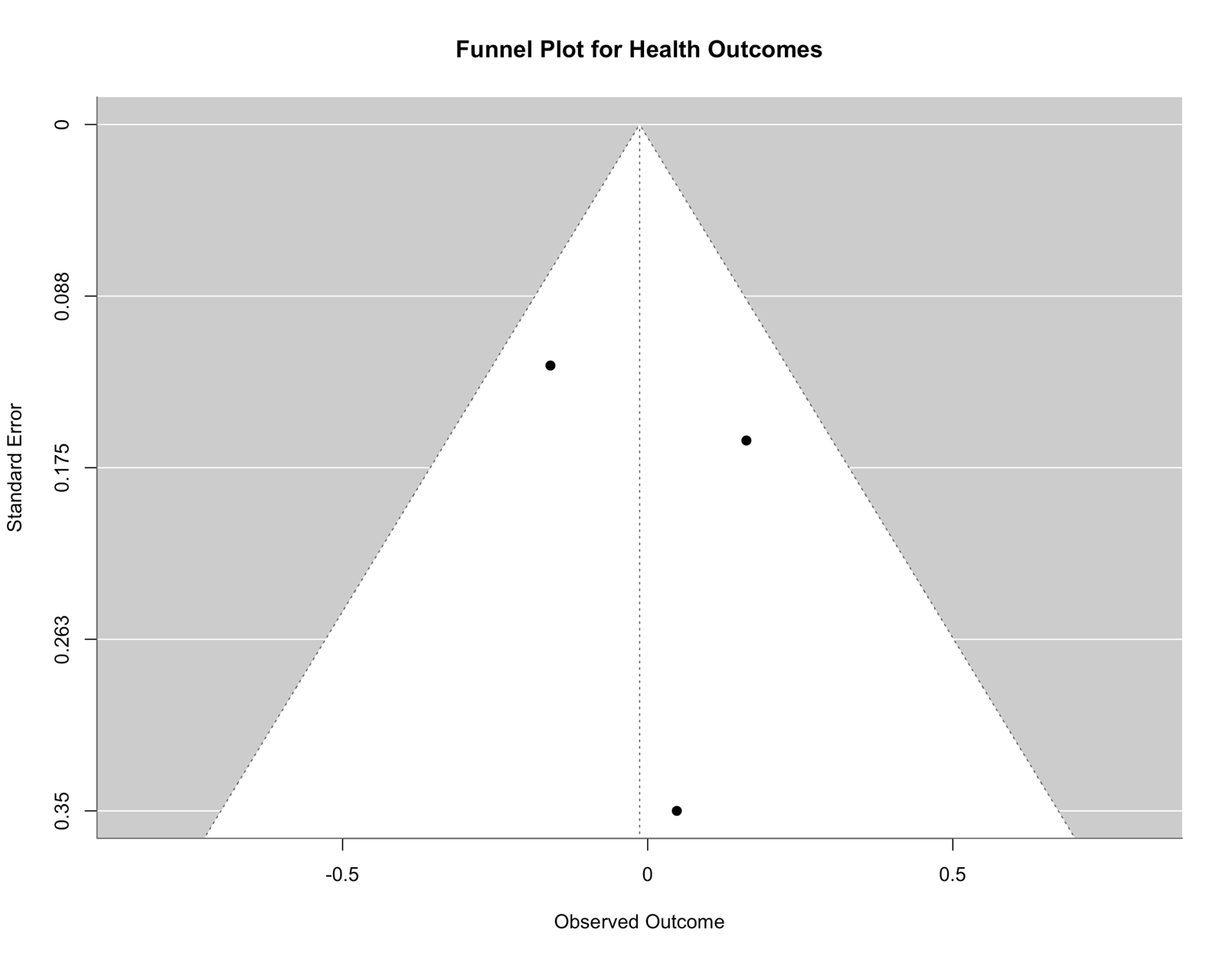

Supplement: Multimedia Appendix 5 [file jmir-v27-e76296-s005.docx]
